# Integrated Genotypic and Phenotypic Determinants Underpin the Environmental Persistence of *Escherichia coli* in Treated Sewage in Japan

**DOI:** 10.1111/1758-2229.70452

**Published:** 2026-10-30

**Authors:** Ida Munfarida, Jant Cres Caigoy, Christian Xedzro, Toshi Shimamoto, Tadashi Shimamoto

**Affiliations:** ^1^ Graduate School of Integrated Sciences for Life Hiroshima University Higashi‐Hiroshima Hiroshima Japan; ^2^ Faculty of Science and Technology Universitas Islam Negeri Sunan Ampel Surabaya Surabaya Indonesia

**Keywords:** antimicrobial resistance, biofilm, *Escherichia coli*, motility, sewage, siderophore, virulence

## Abstract

*Escherichia coli* poses global health risks, affecting human, animal, and environmental systems. To investigate the determinants underpinning its environmental persistence, we conducted an integrated genotypic and phenotypic analysis of 365 *E*. *coli* isolates recovered from treated sewage in Hiroshima, Japan. Isolates were confirmed by *uidA*‐PCR and screened for virulence, antimicrobial resistance (AMR), phylogroup, and siderophore‐associated genes using multiplex PCR. Phenotypic assays assessed biofilm formation on microtiter plates and motility on soft agar. Among the isolates, 13.7% carried one or more virulence genes, with *astA* being the most prevalent (70.0%), followed by *eaeA* (8.0%), *bfpA* (6.0%), and the *aggR*‐*astA* combination (4.0%). AMR genes were detected in 50.0% of isolates, predominantly *bla*_TEM_. The major phylogroups were D (36.0%) and B1 (24.0%). All isolates carried *entA–F* and *tonB*, while *fecA* and *chuA* were the predominant accessory iron‐acquisition genes (52.0%). Phenotypic analysis showed that 92.0% of isolates formed biofilms and 100.0% were motile. *aggR*‐positive isolates exhibited the highest biofilm production, whereas *astA*‐, *bfpA*‐, and *eaeA*‐positive isolates demonstrated greater motility than *ipaH*‐positive isolates. Principal component analysis indicated that biofilm formation was associated with *aggR*, whereas motility showed an opposing trend. Together, these traits contribute to the environmental persistence of *E*. *coli* in treated sewage.

## Graphical abstract

This study integrated genotypic and phenotypic analyses to investigate the persistence of *Escherichia coli* in treated sewage in Japan. Virulence‐positive isolates were dominated by phylogroups D and B1, with *astA* and *bla*_TEM_ as the predominant virulence and resistance determinants. Among accessory siderophore genes, *fecA* and *chuA* were the most prevalent. Biofilm formation was associated with *aggR*, whereas motility showed an opposing trend. These traits may promote persistence of pathogenic *E*. *coli* in aquatic environments and pose a public health risk.

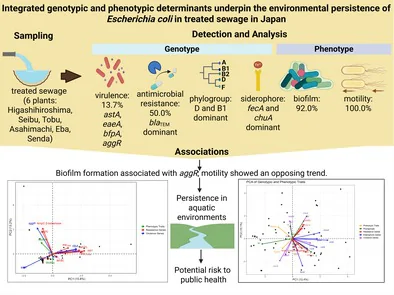

## 1 Introduction

*Escherichia coli* is a Gram‐negative bacterium that commonly inhabits the gastrointestinal tract of humans and other warm‐blooded animals. Most *E*. *coli* strains are harmless commensals that form part of the normal intestinal microbiota. However, certain strains have acquired specific virulence factors that enable them to cause disease and are therefore classified as pathogenic *E*. *coli* (Pepper et al. 2015). These pathogenic strains are a major global health concern, particularly due to their involvement in foodborne illnesses and their capacity to cause severe gastrointestinal and systemic infections (World Health Organization 2015; Singha et al. 2023; Quinn et al. 2025; European Food Safety Authority 2025). Five major pathotypes of diarrheagenic *E*. *coli* (DEC) are recognized: enterohemorrhagic *E*. *coli* (EHEC), enteropathogenic *E*. *coli* (EPEC), enterotoxigenic *E*. *coli* (ETEC), enteroaggregative *E*. *coli* (EAEC), and enteroinvasive *E*. *coli* (EIEC) (Ohmura‐Hoshino et al. 2022). According to the World Health Organization (2024), diarrheal diseases remain a leading cause of death in many regions, particularly in low‐income countries and the African Region, where they consistently ranked among the top 10 causes of death from 2000 to 2021. The distribution of DEC pathotypes varies geographically. For example, EAEC has been reported as the predominant DEC pathotype in Mexico (Van Nederveen and Melton‐Celsa 2025). Nevertheless, ETEC remains a major cause of diarrheal disease worldwide, accounting for an estimated 220 million diarrheal episodes annually, including approximately 75 million episodes among children under 5 years of age (World Health Organization 2021). Another major global concern is Shiga toxin‐producing *E*. *coli* (STEC). Since its identification in 1982, more than 39,787 STEC cases linked to 1343 outbreaks have been reported, with the majority occurring in the Americas, followed by Europe, Asia, and Africa (Alhadlaq et al. 2024). According to recent surveillance data from the Centers for Disease Control and Prevention, 37 outbreaks caused by STEC were reported in the United States in 2024 (Centers for Disease Control and Prevention 2022). In Japan, EHEC, a Shiga toxin‐producing pathotype, remains a notable public health threat. In 2020 alone, 3088 cases were reported nationwide, including 1985 symptomatic infections and multiple outbreaks in nursery schools and community settings (National Institute of Infectious Diseases 2021).

In addition to DEC, extraintestinal pathogenic *E*. *coli* (ExPEC) represents another major public health concern. Unlike intestinal pathotypes, ExPEC strains cause infections outside the gastrointestinal tract, including urinary tract infections, bloodstream infections, and prostatitis (Manges et al. 2019). ExPEC is recognized as one of the leading causes of community—and healthcare‐associated infections worldwide. These pathogenic strains often carry a range of virulence genes, including those encoding adhesins, invasins, and toxins (Sora et al. 2021; Zhang et al. 2025).

The environmental dissemination of *E*. *coli* , particularly through sewage treatment systems, is an emerging concern within the One Health framework, which emphasizes the interconnectedness of human, animal, and environmental health. The environment serves not only as a reservoir which pathogens can survive and multiply but also as a site of ecological interactions and transmission pathways (Centers for Disease Control and Prevention 2022). Sewage treatment plants (STPs) receive a complex mixture of microbial communities, pharmaceutical residues, and host‐associated genetic material, and may therefore serve as reservoirs and potential amplification points for bacteria harbouring clinically relevant traits, such as *E*. *coli* (Cheng et al. 2024; Alfarsi et al. 2025; Malik et al. 2024). *E*. *coli* strains originating from sewage, particularly those harbouring both virulence and resistance traits, pose cross‐ecosystem risks, as they can spread from treated effluents into surrounding water bodies, such as rivers (Yu et al. 2022; Biggel et al. 2023). Their ability to acquire and disseminate genes through horizontal transfer further increases the threat they pose to public and animal health (Hu et al. 2024).

In Japan, where advanced sewage management systems are in place, the microbial quality of treated effluents remains a public health concern, particularly regarding the persistence of *E*. *coli* strains that may retain virulent or environmentally adaptive traits. Although national surveillance has provided extensive data on clinical and hospital‐associated *E*. *coli* , no data are currently available on the genotypic and phenotypic characteristics of *E*. *coli* populations in treated sewage in Japan. Understanding the potential risks posed by environmental isolates requires an integrated approach that examines both their genetic potential and functional behaviors. Genotypic traits, such as phylogenetic group affiliations, virulence genes, resistance genes, and siderophore‐related genes, provide insights into the pathogenic potential and evolutionary backgrounds of *E*. *coli* strains. Phylogenetic grouping (A, B1, B2, C, D, E, and F) is commonly used to infer pathogenic potential, with groups B2 and D frequently linked to ExPEC (Wyrsch et al. 2024; Bhattacharjee et al. 2023). Moreover, phenotypic features such as biofilm formation and motility play crucial roles in bacterial persistence, colonization, and survival in aquatic environments, allowing *E*. *coli* to adhere to surfaces and resist environmental stressors, including disinfectants and antimicrobials (Ballén et al. 2022; Shree et al. 2023). Although motility is not restricted to pathogenic *E*. *coli* , it may influence environmental fitness by facilitating bacterial movement, colonization, and dissemination (Zhao et al. 2023). However, to date, no study in Japan has comprehensively investigated the co‐occurrence of these genotypic and phenotypic traits in *E*. *coli* strains derived from treated sewage, as previous research has predominantly focused on clinical or hospital‐derived isolates.

This study is the first to explore such characteristics in *E*. *coli* isolates collected from treated sewage at six sites in Hiroshima (Higashihiroshima, Seibu, Tobu, Asahimachi, Eba, and Senda), representing both combined (Asahimachi, Eba, Senda) and separate sewer systems (Higashihiroshima, Seibu, and Tobu). In a combined sewer system, sewage (wastewater from homes and buildings) and rainwater are collected in a single pipe and conveyed to a treatment facility. However, during periods of heavy rain, these systems are at risk of overflowing, which can result in the discharge of untreated water into the environment. In contrast, a separate sewer system uses two distinct pipes, with one transporting sewage to a purification center and the other discharging rainwater directly into rivers or the environment. This design is more efficient during storms and reduces the risk of environmental pollution (Kumamoto City Waterworks and Sewerage Bureau 2018). Including sites representing both sewer system types enabled an exploratory comparison of *E*. *coli* populations across different wastewater management settings and provided an opportunity to assess whether the distributions of virulence‐associated traits, antimicrobial resistance (AMR) genes, and siderophore‐associated genes differed between these infrastructure types. The aims of this study were to (i) characterize the virulence, AMR, phylogenetic, and siderophore‐associated profiles of wastewater‐derived *E*. *coli* ; (ii) assess phenotypic traits associated with environmental persistence, including biofilm formation and motility; and (iii) investigate the relationships among these genotypic and phenotypic characteristics. Through this integrated approach, the study provides insight into the ecological characteristics and environmental persistence of wastewater‐derived *E*. *coli* and supports the broader application of One Health principles in environmental microbiology.

## 2 Experimental Procedures

### 2.1 Description of the Sampling Sites

*E*. *coli* isolates were collected from sewage treatment water in Hiroshima Prefecture, Japan. The sampling sites were Higashihiroshima, Seibu, Tobu, Asahimachi, Eba, and Senda (Figure 1). These sites were selected to represent two distinct types of sewer systems: combined sewer systems, which transport both domestic wastewater and stormwater through a single pipeline (Asahimachi, Eba, and Senda), and separate sewer systems, which manage these flows independently through separate pipelines (Higashihiroshima, Seibu, and Tobu).

**FIGURE 1:**
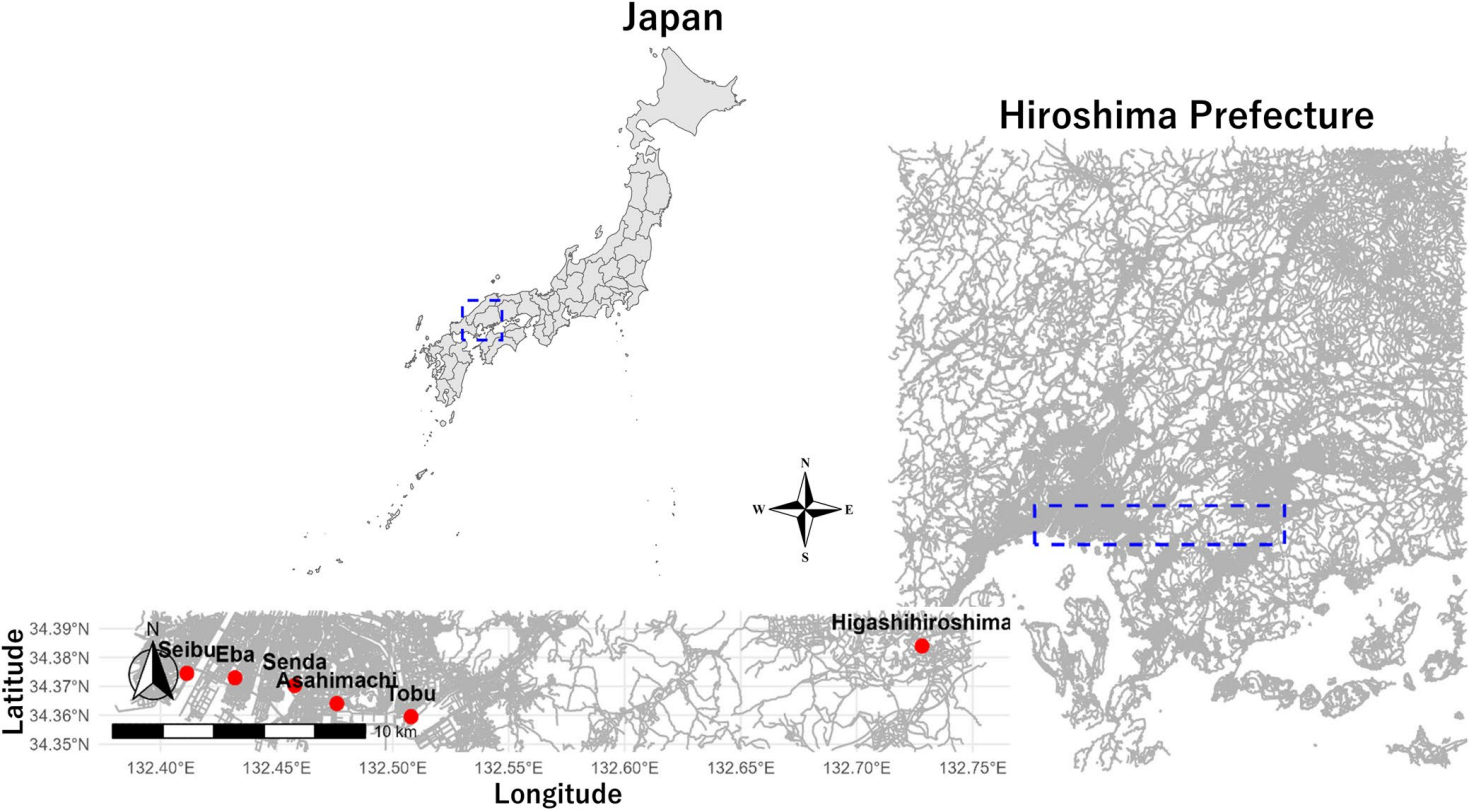
Geographic locations of the six municipal sewage treatment plants (STPs) sampled in Hiroshima Prefecture, Japan. The map of Japan (upper left) shows the location of Hiroshima Prefecture, and the map of Hiroshima Prefecture (upper right) shows the study area. Blue dashed rectangles indicate the areas enlarged in the subsequent maps. Red circles in the detailed map indicate the six sampling locations: Seibu, Eba, Senda, Asahimachi, Tobu, and Higashihiroshima. All maps were generated in R using Natural Earth public‐domain geographical data.

### 2.2 Sampling Procedure

Between February 2017 and December 2024, sewage treatment water samples were collected from the final effluents of six municipal STPs in Hiroshima Prefecture, Japan (Asahimachi, Eba, Senda, Seibu, Tobu, and Higashihiroshima) as part of an ongoing wastewater monitoring program. These facilities are centralized public sewerage treatment systems serving municipal populations and are distinct from decentralized onsite treatment systems such as Johkasou. Samples were collected from the final treated effluent after completion of the wastewater treatment process and prior to environmental discharge. Across all facilities, chlorination was employed as the final disinfection step before effluent discharge, as indicated by the respective treatment‐process information provided by Hiroshima City, Higashihiroshima City, and the Hiroshima Prefecture Sewerage Public Corporation (Hiroshima City Sewerage Bureau 2026; Higashihiroshima City 2026; Hiroshima Prefecture Sewerage Public Corporation 2026). The *E*. *coli* isolates analysed in this study comprised both archived isolates recovered from earlier monitoring years and newly collected isolates obtained during 2023–2024. Sampling procedures followed the guidelines outlined by the Department of Water Affairs and Forestry (2000) and the Environment Protection Authority (2007). Briefly, samples were obtained using sterile 1‐L polyethylene bottles, each representing a single time‐point collection. All samples were transported to the laboratory at ambient temperature and processed on the same day. Seasonal classification, Tsuyu status, and rainfall‐related variables were determined from the sampling dates and locations. Seasons were classified as spring (March–May), summer (June–August), autumn (September–November), and winter (December–February), while Tsuyu status was classified as rainy season (June–July) or non‐rainy season. Corresponding meteorological data, including temperature and precipitation, were retrieved from Open‐Meteo using the sampling coordinates and dates and were processed in R (version 4.4.2; R Core Team 2024; Open‐Meteo 2026).

### 2.3 Isolation of *E*. *coli*

*E*. *coli* was isolated using the membrane filtration method described by Cappuccino and Welsh (2020). Two enrichment strategies were applied: (1) non‐selective enrichment using Luria‐Bertani (LB) broth (Nacalai Tesque, Japan), and (2) selective enrichment with LB broth containing 100 μg/mL ampicillin. Both cultures were incubated at 37°C for 22–24 h. After incubation, the cultures were streaked onto the following selective media: CHROMagar ECC (CHROMagar, France) and MacConkey agar (Pearlcore MacConkey, Japan) with colistin (2 μg/mL) for non‐selective cultures, and CHROMagar ECC with ampicillin (100 μg/mL) and MacConkey agar with meropenem (2 μg/mL) for selective cultures.

### 2.4 DNA Extraction

DNA was extracted following the protocol described by Dashti et al. (2009) with some modifications. Briefly, pure cultures of *E*. *coli* isolates were grown in 5 mL of nutrient broth and incubated overnight at 37°C with shaking at 160 rpm. Cultures were then adjusted to an optical density at 600 nm (OD₆₀₀) of 0.1, and cells were pelleted by centrifugation at 13,000 × *g* for 3 min. The pellet was resuspended in 500 μL of Tris‐EDTA (TE) buffer and boiled at 100°C for 10 min. After centrifugation at 13,000 × *g* for 5 min, the supernatant containing DNA was collected and stored at −20°C until use.

### 2.5 Detection of *E*. *coli* by PCR

*E*. *coli* isolates were confirmed by a single PCR targeting the *uidA* gene, following the method described by Jenifer and Sathiyamurthy (2020), with minor modifications. Primers EcuidA‐F (5′‐GGGAATGGTGATTACCGACGAAAACGGC‐3′) and EcuidA‐R (5′‐ACAGACGCGTGGTTACAGTCTTGCG‐3′) amplified a 171‐bp product. *Salmonella enterica* serovar Typhimurium strain LT2, which was used as a negative control, and ETEC strain 0509271, which was used as a positive control. Both strains were obtained from the Laboratory of Food Microbiology and Hygiene at Hiroshima University. These strains were originally described by Caigoy et al. (2024) and Kusalaruk et al. (2024), and were used as reference controls in this study.

### 2.6 Detection of Selected Virulence Genes by Multiplex PCR (mPCR)

Major virulence determinants associated with DEC were detected using mPCR. Targeted genes were selected based on the classification system described by Ohmura‐Hoshino et al. (2022), covering five major DEC pathotypes: ETEC, EPEC, EAEC, EIEC, and EHEC. The thermal cycling conditions were as described by Ohmura‐Hoshino et al. (2022), with minor modifications. A panel of *E*. *coli* reference strains was pre‐validated and used as positive controls. Positive controls included the following strains: ETEC strain 0509271 (positive for *astA*, *sth*, *elt*); EHEC strain RIMD 0509891 (positive for *astA*, *eaeA*, *stx*_*1*_, *stx*_*2*_); EPEC strain 0509829 (positive for *bfpA* and *eaeA*); EAEC strain 0591633 (positive for *astA* and *aggR*); and EIEC strain 0591045 (positive for *invE* and *ipaH*). Positive control strains were obtained from the Laboratory of Food Microbiology and Hygiene at Hiroshima University. These strains were originally described by Kusalaruk et al. (2024) and were used as reference controls. In addition, *E*. *coli* CA‐1‐3, which was isolated in this study and confirmed to lack all targeted virulence genes, was used as the negative control. To ensure amplification efficiency and monitor potential reaction failures or artefacts, an external positive control comprising pooled DNA supernatants from the five DEC reference strains was included in every run and processed in parallel with the test samples. An internal control (IC) targeting the *E*. *coli* 16S rRNA gene was included in all 365 test reactions using universal primers 27F and 1492R to prevent false‐negative results. The approximately 1500‐bp 16S rRNA fragment was amplified using the universal primers 27F (5′‐AGAGTTTGATCMTGGCTCAG‐3′) and 1492R (5′‐CGGYTACCTTGTTACGACTT‐3′), as described by Xedzro, Shimamoto, and Shimamoto (2023). Additionally, single PCR assays were performed, with minor modifications to the thermal cycling conditions, to confirm the presence of specific virulence genes detected in the mPCR. The primer sets used to detect the targeted virulence genes are listed in Table S1.

### 2.7 Resistance Gene Identification

A series of mPCR assays was used to detect a broad range of AMR genes in *Escherichia coli* isolates recovered from treated sewage. The eicosa‐plex system simultaneously targets 20 markers: the 16S rRNA gene as an amplification control; eight non‐AmpC AMR determinants or gene groups (*bla*_OXA‐1_, *bla*_SHV_, *bla*_TEM_, *bla*_CTX‐M_, *bla*_OXA‐2_, *bla*_OXA‐5_, *aac*(*6′*)*‐Ib*, and *bla*_OXA‐9_); eight AmpC β‐lactamase gene groups (*bla*_CMY/LAT_, *bla*_MIR/ACT_, *bla*_DHA_, *bla*_MOX_, *bla*_FOX_, *bla*_PDC_, *bla*_ACC_, and *bla*_ADC_); and three class integron integrase genes (*intI1*, *intI2*, and *intI3*). The octa‐plex system was used to differentiate the eight AmpC β‐lactamase gene groups, whereas the CR major‐plex system was used to detect five major carbapenemase gene groups: *bla*_KPC_, *bla*_NDM_, *bla*_OXA‐48_, *bla*_VIM_, and *bla*_IMP_. All three mPCR systems were performed according to the protocols described by Soliman et al. (2023). PCR was used to detect *mcr* genes conferring colistin resistance. Two additional mPCR assays were used to detect colistin‐resistance genes, targeting *mcr‐1* to *mcr‐5* and *mcr‐6* to *mcr‐10*, following the methods of Jousset et al. (2019), Borowiak et al. (2020), and Wang et al. (2020), with minor modifications. The tetracycline resistance genes *tet*(A), *tet*(B), *tet*(C), *tet*(D), *tet*(E), and *tet*(G) were screened using the method described by Ng et al. (2001), with minor modifications. An internal control targeting the *E*. *coli* 16S rRNA gene (primers 515F and 1492R) was included in all reactions to prevent false‐negative results. Single PCR assays were performed under the same thermal cycling conditions to confirm the specific genes detected by mPCR. Positive control strains for resistance gene detection were obtained from the Laboratory of Food Microbiology and Hygiene at Hiroshima University. These strains were originally described by Soliman et al. (2023) and were used as reference controls in this study. The primer details for all assays are provided in Table S2.

### 2.8 Phylogenetic Group Determination

Phylogenetic classification of *E*. *coli* isolates was performed using the revised PCR‐based scheme developed by Clermont et al. (2013) with minor modifications. This method employs quadruplex PCR to detect four genetic markers (*arpA*, *chuA*, *yjaA*, and the DNA fragment TspE4.C2) to differentiate among the major phylogroups A, B1, B2, C, D, E, F, and cryptic clades. Positive control strains for phylogenetic analysis were obtained from the Laboratory of Food Microbiology and Hygiene at Hiroshima University. These strains were originally described by Xedzro, Kimura, et al. (2023) and were used as reference controls in this study. As a negative control, TE buffer was used in place of template DNA. The primer sequences are listed in Table S3.

### 2.9 Siderophore Gene Detection

Thirteen genes involved in siderophore biosynthesis and transport were screened in *E*. *coli* isolates using mPCR. Six genes associated with enterobactin biosynthesis (*entA*, *entB, entC*, *entD*, *entE*, and *entF*) were targeted using two multiplex panels with primer sequences and protocols adapted from Khazaal et al. (2024) with minor modifications. The remaining seven siderophore‐related genes were grouped into three additional mPCR panels based on the protocol described by Rahmani et al. (2020) with minor modifications: Panel 1 (*iroN*, *iutA*, *fecA*), Panel 2 (*fyuA*, *sitA*), and Panel 3 (*chuA*, *tonB*). Strains previously confirmed to carry the targeted siderophore genes were validated before sample testing and used as positive controls. An internal control targeting the *E*. *coli* 16S rRNA gene was included in all reactions to prevent false‐negative results, using primers 27F and 1492R. Single PCR assays were performed under the same thermal cycling conditions to confirm the genes detected by mPCR. Details of the primer sequences and expected amplicon sizes are listed in Table S4.

### 2.10 Biofilm Formation Assay

Biofilm formation by *E*. *coli* isolates was assessed using a 96‐well microtiter plate assay based on the method described by Coffey and Anderson (2014), with minor modifications. Overnight LB cultures were diluted 1:100, and 200 μL of each diluted culture was added to each well. Plates were incubated statically at 37°C for 24 h. The wells were washed to remove nonadherent cells, stained with 0.1% crystal violet for 10 min, rinsed, and treated with 95% ethanol to solubilize the stain. Biofilm biomass was quantified by measuring optical density at 570 nm. Each biofilm assay was performed in three independent experiments, and the mean value was used for analysis. Uninoculated LB broth served as the negative control. To interpret the results, the cut‐off optical density value (ODc) was calculated as three standard deviations above the mean OD of the negative control. Isolates were categorized based on their OD₅₇₀ values relative to the ODc. Isolates were classified as non‐biofilm producers (OD ≤ ODc), weak biofilm producers (ODc <OD ≤ 2ODc), moderate biofilm producers (2ODc < OD ≤ 4ODc), or strong biofilm producers (OD > 4ODc).

### 2.11 Motility Assay

The motility of *E*. *coli* isolates was evaluated using a standard soft‐agar assay, following the protocol described by Partridge and Harshey (2021) with minor modifications. Semi‐solid motility agar was prepared by supplementing LB broth with 0.3% agar. Each isolate was vertically stab‐inoculated into the center of a motility agar tube using a sterile toothpick. Following inoculation, the tubes were incubated at 37°C for 12 h under aerobic conditions. After incubation, bacterial motility was assessed based on the growth patterns within the agar. Motile isolates exhibited diffuse, radiating growth extending from the stab line, whereas non‐motile isolates displayed restricted growth limited to the original point of inoculation.

### 2.12 In Silico Detection of Virulence and Siderophore Genes

To compare our experimental findings with those of known *E*. *coli* strains, we retrieved the genome sequences of 50 representative *E*. *coli* isolates from the EnteroBase database (EnteroBase 2025; https://enterobase.warwick.ac.uk). These strains were selected because they represent diverse phylogenetic backgrounds and ecological niches. We performed an *in silico* screening to detect the presence of specific virulence genes and siderophore‐associated genes. The 16S rRNA gene was used as the internal positive control. Primer sequences for each target gene were compiled from published literature and formatted as forward and reverse oligonucleotide pairs. Genome sequences were screened using the Biostrings package in R (version 4.4.2; R Core Team 2024), which allowed pattern matching with a configurable mismatch tolerance. Each genome was screened for exact matches (zero mismatches) and near‐exact matches (one mismatch) to accommodate minor sequence variations. An R script was developed to automate this process.

### 2.13 Statistical Analysis

All statistical analyses were conducted using R software (version 4.4.2). Statistical significance was set at *p* < 0.05. Associations between categorical variables were evaluated using chi‐square tests or Fisher's exact tests, as appropriate, whereas Spearman's rank correlation was used to evaluate relationships between numerical variables. Nonparametric analyses included the Mann–Whitney U test for comparisons between two groups and the Kruskal–Wallis test followed by Bonferroni‐corrected post hoc comparisons for comparisons among multiple groups. Temporal differences in the prevalence of virulence‐positive and AMR‐positive isolates among sampling years were assessed using Fisher's exact tests. Where applicable, multiple comparisons were adjusted using the Benjamini–Hochberg procedure. Principal component analysis (PCA) was used to explore the data structure, and canonical correlation analysis (CCA) was used to assess multivariate associations.

## 3 Results

### 3.1 Isolation and Identification of Bacterial Isolates

A total of 365 *E*. *coli* isolates were recovered across various sampling locations, dates, and sewage treatment systems (Table S5), and their identity was confirmed by PCR targeting the *uidA* gene (Figure S1). The isolate counts varied over time, with prominent peaks observed on July 22, 2024 (*n* = 34), October 31, 2023 (*n* = 26), and May 8 and July 19, 2023 (*n* = 25 each), suggesting temporally elevated *E*. *coli* presence during these periods. Sampling was conducted under a wide range of seasonal and weather conditions. The highest proportion of isolates was recovered during summer (35.6%, 130/365), followed by spring (30.7%, 112/365), autumn (23.3%, 85/365), and winter (10.4%, 38/365). Based on the Japanese rainy season (Tsuyu; June–July), 35.6% (130/365) of isolates were collected during the rainy season, whereas 64.4% (235/365) were recovered during the non‐rainy season. Nearly half of all isolates (49.9%, 182/365) were collected on dry days, while 50.1% (183/365) were recovered during rainfall events, including very light (19.5%, 71/365), light‐to‐moderate (25.5%, 93/365), and heavy rainfall (5.2%, 19/365) conditions (Table S5). Geographically, the highest numbers of isolates were recovered from Eba (*n* = 98) and Asahimachi (*n* = 86), with both locations yielding significantly more isolates than Higashihiroshima and Tobu. The majority were collected from areas with a combined sewage treatment system (*n* = 246), significantly more than from areas with a separate system (*n* = 119) (Table S6).

### 3.2 Virulence Gene Detection

Of the 365 confirmed *E*. *coli* isolates, 50 (13.7%) harboured one or more virulence genes, as summarized in Table 1 (see PCR documentation in Figure S2). Among the 50 isolates, the most frequently detected gene was *astA*, identified in 70.0% of the strains, which were categorized as other diarrheagenic *E*. *coli* (ODEC) according to Kashima et al. (2021). The virulence genes *eaeA* and *bfpA* were detected in 8.0% and 6.0% of the isolates, respectively. Based on the presence of *eaeA*, the isolates were classified as atypical EPEC (*bfpA*−) or typical EPEC (*bfpA*+) (Lee et al. 2024; McDougall et al. 2023). The remaining *bfpA*‐positive but *eaeA*‐negative isolates were reported as *bfpA*‐positive isolates. The *aggR–astA* gene combination, associated with hetero‐pathogenic strains (tEAEC/ODEC), was detected in 6.0% of the isolates. Two isolates carrying *ipaH* were classified as EIEC. A single isolate carrying *aggR* alone was classified as typical EAEC (Santos et al. 2020). One isolate harboured both *eaeA* and *bfpA*, indicating an EPEC profile (Ohmura‐Hoshino et al. 2022). In addition, one isolate carrying *elt* was identified as ETEC (Santos et al. 2020). Furthermore, a single isolate harboured *stx*_*2*_ and was classified as STEC (Lee et al. 2024). To assess potential environmental associations with virulence status, the distributions of virulence‐positive and virulence‐negative isolates were compared across seasons and weather conditions. Fisher's exact tests revealed no significant association between virulence status and season, the Japanese rainy season, rainy versus dry conditions, or rainfall category. Similarly, Wilcoxon rank‐sum tests showed no significant differences in mean daily temperature or precipitation amount between virulent and non‐virulent isolates. In contrast, temporal analysis revealed a significant association between virulence status and sampling year. Post hoc year‐versus‐all‐other‐years comparisons showed that virulence‐positive isolates were significantly more prevalent in 2023 (20.0%), whereas 2021 exhibited a significantly lower prevalence (6.1%). A chi‐squared test revealed a significantly higher number of virulent *E*. *coli* isolates in areas utilizing a combined sewage treatment system compared to those with a separate system. The distribution of virulence‐positive isolates also differed significantly among sampling sites, with the highest numbers recorded in Eba, Senda, and Asahimachi (Table S7).

**TABLE 1:** Distribution of virulence genes and corresponding pathotypes among *E*. *coli* isolates (*n* = 50) carrying one or more virulence genes.

No	Virulence gene	Number of isolates	Percentage (%)	Pathotype
1	*astA*	35	70	ODEC
2	*eaeA*	4	8	Atypical EPEC
3	*bfpA*	3	6	*bfpA*‐positive isolates
4	*aggR, astA*	2	4	Hetero‐pathogen (EAEC/ODEC)
5	*ipaH*	2	4	EIEC
6	*aggR*	1	2	Typical EAEC
7	*eaeA, bfpA*	1	2	Typical EPEC
8	*elt*	1	2	ETEC
9	*stx*_*2*_	1	2	STEC
Total		50	100	

### 3.3 Resistance Gene Distribution

Among the 50 virulence‐positive *E*. *coli* isolates, 25 (50.0%) harboured one or more AMR genes (Table 2; see PCR documentation in Figure S3). A heatmap was generated to examine associations between virulence genes and AMR gene groups among the *E*. *coli* isolates (Figure 2a). Although the virulence gene *astA* was frequently detected among isolates carrying extended‐spectrum β‐lactamase (ESBL) genes, no statistically significant global association was observed between virulence profiles and resistance groups. To assess specific gene‐pair relationships, a volcano plot was generated from Individual 2 × 2 contingency‐table tests for each virulence–resistance‐group combination (Figure 2b). Among the pairs tested, the association between *aggR* and AmpC β‐lactamase/*tet*(A) exhibited the largest effect size and the lowest *p* value. However, this association did not reach statistical significance. Similarly, comparison of isolates recovered from combined and separate sewer systems revealed no significant differences in the prevalence of AMR genes. In contrast, temporal analysis revealed a significant association between AMR status and sampling year. Post hoc year‐versus‐all‐other‐years comparisons showed that AMR‐positive isolates were significantly more prevalent in 2023 (12.3%), whereas no other years remained significant after correction for multiple comparisons (Table S8).

**TABLE 2:** Summary of resistance gene combinations among *E*. *coli* isolates.

No	Resistance gene	Number of isolates	Percentage (%)
1	No resistance genes detected	25	50.0
2	*bla*_TEM_	11	22.0
3	*bla*_TEM_, *intI1*	4	8.0
4	*bla*_CTX‐M_	2	4.0
5	*bla*_TEM_, *tet*(B)	2	4.0
6	AmpC β‐lactamase, *tet*(A)	1	2.0
7	*bla*_TEM_, *bla*_CTX‐M_, *intl1*	1	2.0
8	*bla*_TEM_, *bla*_CTX‐M_, *intl1*, *tet*(B)	1	2.0
9	*bla*_TEM_, *bla*_CTX‐M_, *intl1*, *tet*(D)	1	2.0
10	*bla*_TEM_, *bla*_DHA_	1	2.0
11	*bla*_TEM_, *tet*(A)	1	2.0
Total		50	100.0

**FIGURE 2:**
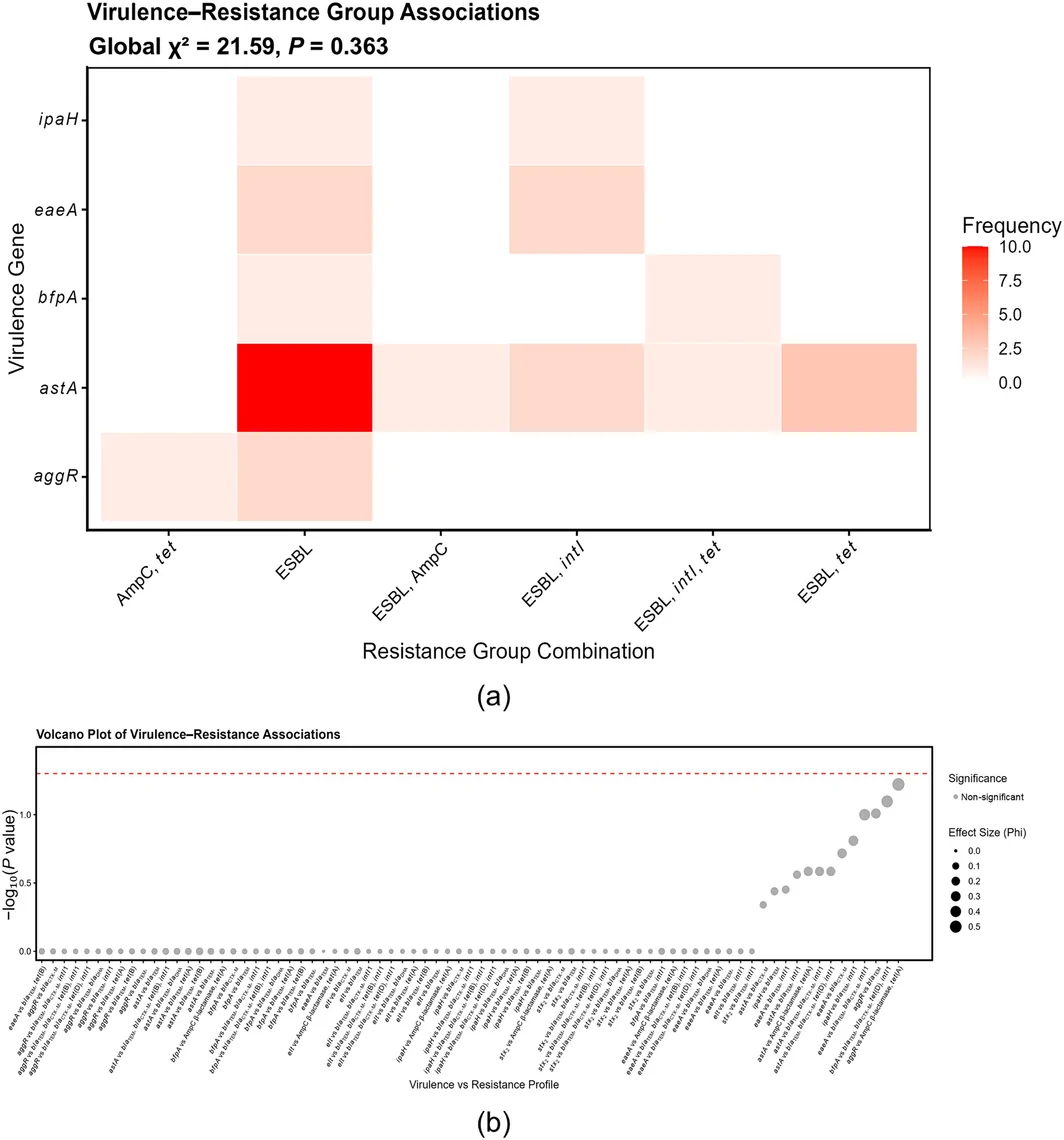
Associations between virulence genes and antimicrobial resistance (AMR) profiles in *Escherichia coli* . (a) Heatmap showing the frequency of isolates carrying each virulence gene across different combinations of resistance groups. Increasing colour intensity indicates a higher frequency of isolates. The overall association between virulence genes and resistance‐group combinations was not statistically significant (*p* = 0.363). (b) Volcano plot showing the individual associations between virulence genes and resistance profiles. The vertical axis represents −log₁₀(*p* value), and point size represents the strength of association measured using the phi coefficient. The red dashed line indicates the significance threshold of *p* = 0.05; all associations were non‐significant. ESBL, extended‐spectrum β‐lactamase; AmpC, AmpC β‐lactamase; *intI*, integron integrase gene; *tet*, tetracycline‐resistance gene.

### 3.4 Phylogenetic Group Determination

A total of 50 *E*. *coli* isolates harbouring virulence genes were analysed for phylogenetic group distribution. The majority of the isolates belonged to phylogroup D (*n* = 18, 36.0%), followed by B1 (*n* = 12, 24.0%), B2 (*n* = 11, 22.0%), A (*n* = 5, 10.0%), and the unknown group (*n* = 2, 4.0%). Phylogroup F and the clade group each contained one isolate (*n* = 1, 2.0% each) (Table S9) (see PCR documentation in Figure S4). The *astA* gene was most frequently detected in phylogroups D (*n* = 13) and B1 (*n* = 9). Other virulence genes, including *eaeA*, *bfpA*, *ipaH* and *aggR* were distributed across several phylogroups. Fisher's exact test with Monte Carlo simulation showed no statistically significant relationship between virulence genes and phylogroups, indicating that the distribution of virulence genes was independent of phylogenetic grouping (Table S9).

### 3.5 Siderophore Gene Distribution

The *ent* gene cluster (*entA*–*entF*) and *tonB* were detected in all isolates (100%). Among the accessory siderophore genes, *fecA* and *chuA* were the most prevalent (52.0% each), followed by *sitA* (48.0%), *fyuA* (46.0%), *iroN* (22.0%), and *iutA* (14.0%) (Table 3) (see PCR documentation in Figure S5). These genes were generally distributed similarly between the virulent and nonvirulent *E*. *coli* groups, particularly the *ent* cluster and *tonB*. The Mann–Whitney U test revealed no significant difference in the total number of siderophore genes between virulent and nonvirulent isolates (Figure 3a). Likewise, Fisher's exact tests showed no significant differences in the prevalence of individual siderophore genes between the two groups (Figure 3b), and no significant association was observed between siderophore gene carriage and sewer system type among virulence gene‐positive isolates (Table S8).

**TABLE 3:** Distribution of siderophore‐associated genes among *E*. *coli* isolates (*n* = 50).

No	Siderophore gene	Number of isolates	Percentage (%)
1	*entA*	50	100.0
2	*entB*	50	100.0
3	*entC*	50	100.0
4	*entD*	50	100.0
5	*entE*	50	100.0
6	*entF*	50	100.0
7	*tonB*	50	100.0
8	*fecA*	26	52.0
9	*chuA*	26	52.0
10	*sitA*	24	48.0
11	*fyuA*	23	46.0
12	*iroN*	11	22.0
13	*iutA*	7	14.0

**FIGURE 3:**
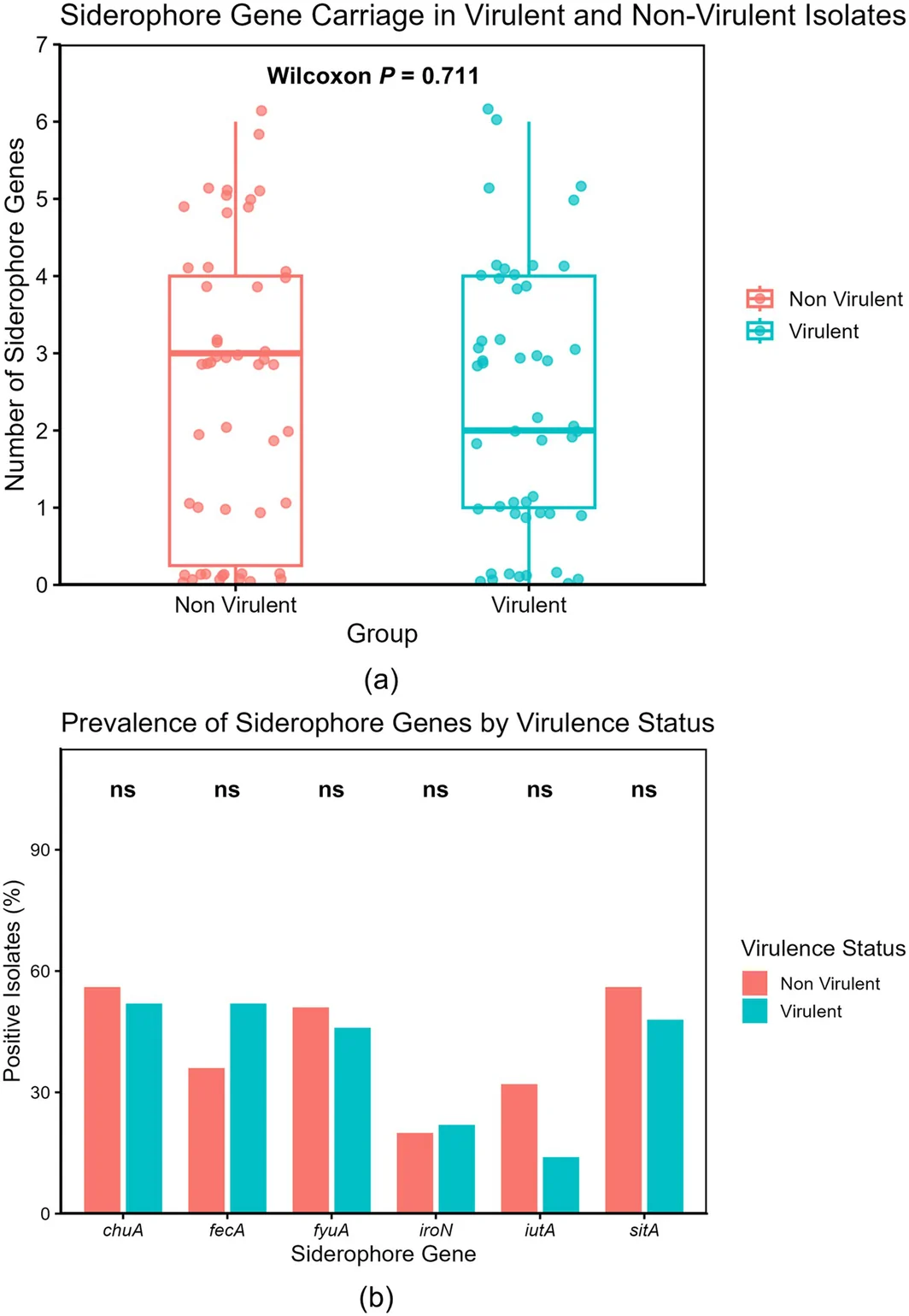
Distribution and prevalence of siderophore genes among virulent and non‐virulent *Escherichia coli* isolates. (a) Number of siderophore genes detected in individual isolates. Points represent individual isolates, centre lines indicate medians, boxes represent the interquartile ranges, and whiskers extend to 1.5 times the interquartile range. No significant difference was observed between the two groups using the Wilcoxon rank‐sum test (*p* = 0.711). (b) Percentages of isolates positive for the additional siderophore genes *chuA*, *fecA*, *fyuA*, *iroN*, *iutA*, and *sitA*. “ns” indicates a non‐significant difference between the virulent and non‐virulent isolates (*p* ≥ 0.05).

### 3.6 In Silico Analysis Results

Fifty *E*. *coli* isolates from diverse source niches were screened *in silico* for selected virulence and iron‐acquisition genes (Table S10). For source‐based comparisons, human and stool isolates were grouped as human‐derived isolates (*n* = 17), whereas livestock, poultry, and swine isolates were grouped as food‐producing animals (*n* = 14). The remaining categories comprised wild‐animal (*n* = 5), food (*n* = 7), and environmental isolates (*n* = 7). Among the virulence genes, *eaeA* and *stx*_*2*_ were the most frequently detected, occurring in 20% (10/50) and 16% (8/50) of the isolates, respectively. However, no significant differences were observed in the distribution of virulence genes among source niches. Among the siderophore‐associated genes, *entA* was the most prevalent, occurring in 68% (34/50) of the isolates, followed by *tonB* in 38% (19/50), and *chuA* and *fecA* in 26% (13/50) each. The prevalence of *entA* was highest among human‐derived isolates (82.4%), followed by food isolates (71.4%), food‐producing‐animal isolates (64.3%), wild‐animal isolates (60.0%), and environmental isolates (42.9%). The prevalence of *chuA* was highest among food isolates (42.9%) and wild‐animal isolates (40.0%). In contrast, *fecA* was most prevalent among human‐derived isolates (47.1%), compared with food‐producing‐animal isolates (21.4%), wild‐animal isolates (20.0%), food isolates (14.3%), and environmental isolates (0%). Although *fecA* showed a moderate association with source niche (Cramér's V = 0.377), this association was not statistically significant (*p* = 0.130), and none of the other investigated gene–niche relationships reached statistical significance (Table S10).

### 3.7 Phenotypic Characterization of *E*. *coli* Isolates

To examine phenotypic traits potentially associated with environmental persistence, biofilm formation, and motility assays were performed.

#### 3.7.1 Biofilm Formation Assay

Among the 50 isolates analysed, 92.0% (*n* = 46) were classified as biofilm producers. Of the 46 biofilm‐producing isolates, 33 (71.7%) were classified as weak, 9 (19.6%) as moderate, and 4 (8.7%) as strong biofilm producers (Table S11). Biofilm formation was significantly greater in virulent isolates than in nonvirulent isolates. Biofilm formation also differed significantly among virulence‐gene groups, with a moderate effect size. Subsequent comparisons showed that *aggR*‐positive isolates exhibited significantly greater biofilm formation than the specified comparison group or groups (Figure 4a,b). Although biofilm formation differed among some virulence‐gene groups, no significant differences were detected among phylogenetic groups. Similarly, biofilm formation was not significantly associated with the presence of AMR or siderophore‐associated genes (Table S12).

**FIGURE 4:**
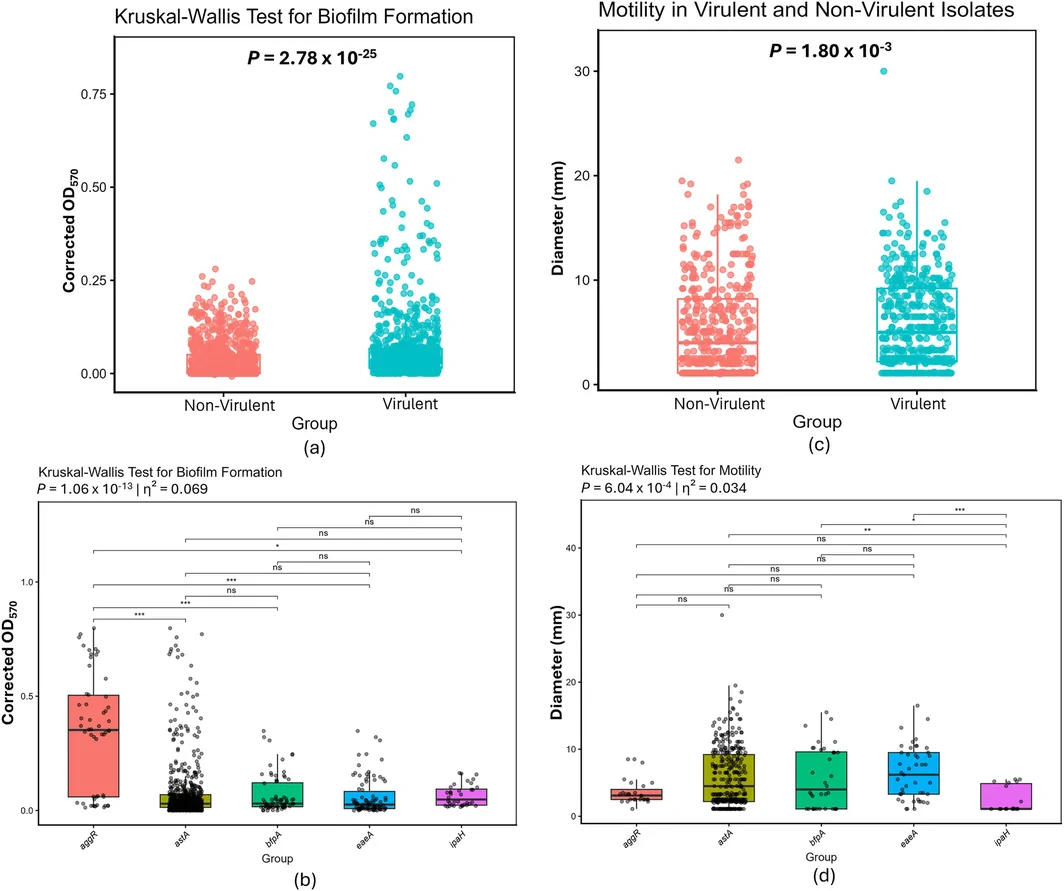
Biofilm formation and motility of *Escherichia coli* isolates according to virulence status and detected virulence genes. (a) Corrected optical density at 570 nm (OD₅₇₀), representing biofilm formation, in non‐virulent and virulent isolates (Kruskal–Wallis test, *p* = 2.78 × 10^−25^). (b) Biofilm formation among isolates carrying *aggR*, *astA*, *bfpA*, *eaeA*, or *ipaH* (Kruskal–Wallis test, *p* = 1.06 × 10^−13^, *η* ^2^ = 0.069). (c) Motility diameters of non‐virulent and virulent isolates (Kruskal–Wallis test, *p* = 1.80 × 10^−3^). (d) Motility diameters among isolates carrying *aggR*, *astA*, *bfpA*, *eaeA*, or *ipaH* (Kruskal–Wallis test, *p* = 6.04 × 10^−4^, *η* ^2^ = 0.034). Points represent individual observations, centre lines indicate medians, boxes represent interquartile ranges, and whiskers extend to 1.5 times the interquartile range. Brackets indicate pairwise comparisons: Ns, not significant; *p < 0.05; **p < 0.01; and ***p < 0.001.

#### 3.7.2 Motility Assay

All 50 virulent *E*. *coli* isolates (100.0%) were motile (Table S13). Isolates carrying any virulence gene demonstrated significantly higher motility compared to virulence‐negative isolates, indicating a strong association between virulence and motility. Motility also varied significantly among virulence‐gene groups; although the effect size was small. Pairwise comparisons revealed that *astA*‐, *bfpA*‐, and *eaeA*‐positive isolates exhibited significantly greater motility than *ipaH*‐positive isolates (Figure 4c,d). In contrast, no significant differences in motility were detected according to phylogroup or the presence of AMR or siderophore‐associated genes. Furthermore, motility was not significantly associated with biofilm formation; although *eaeA—*and *aggR*‐positive isolates showed a tendency toward an inverse relationship between biofilm formation and motility (Table S14).

### 3.8 Genotypic and Phenotypic Interplay in *E*. *coli*

PCA and network correlation analysis were performed to explore the relationship between genotypic markers and phenotypic traits. The first PCA, which included AMR genes, virulence genes, and phenotypic traits, showed a clear separation between motility and biofilm formation (Figure 5a). The *aggR* gene was positioned closer to biofilm formation and farther from motility, suggesting a positive association with biofilm formation and an inverse association with motility. In the second PCA (Figure 5b), several siderophore genes, particularly *iutA*, *iroN*, and *fecA*, showed an association with phylogroup D, suggesting that isolates belonging to this phylogenetic group tended to harbour these siderophore‐associated traits.

**FIGURE 5:**
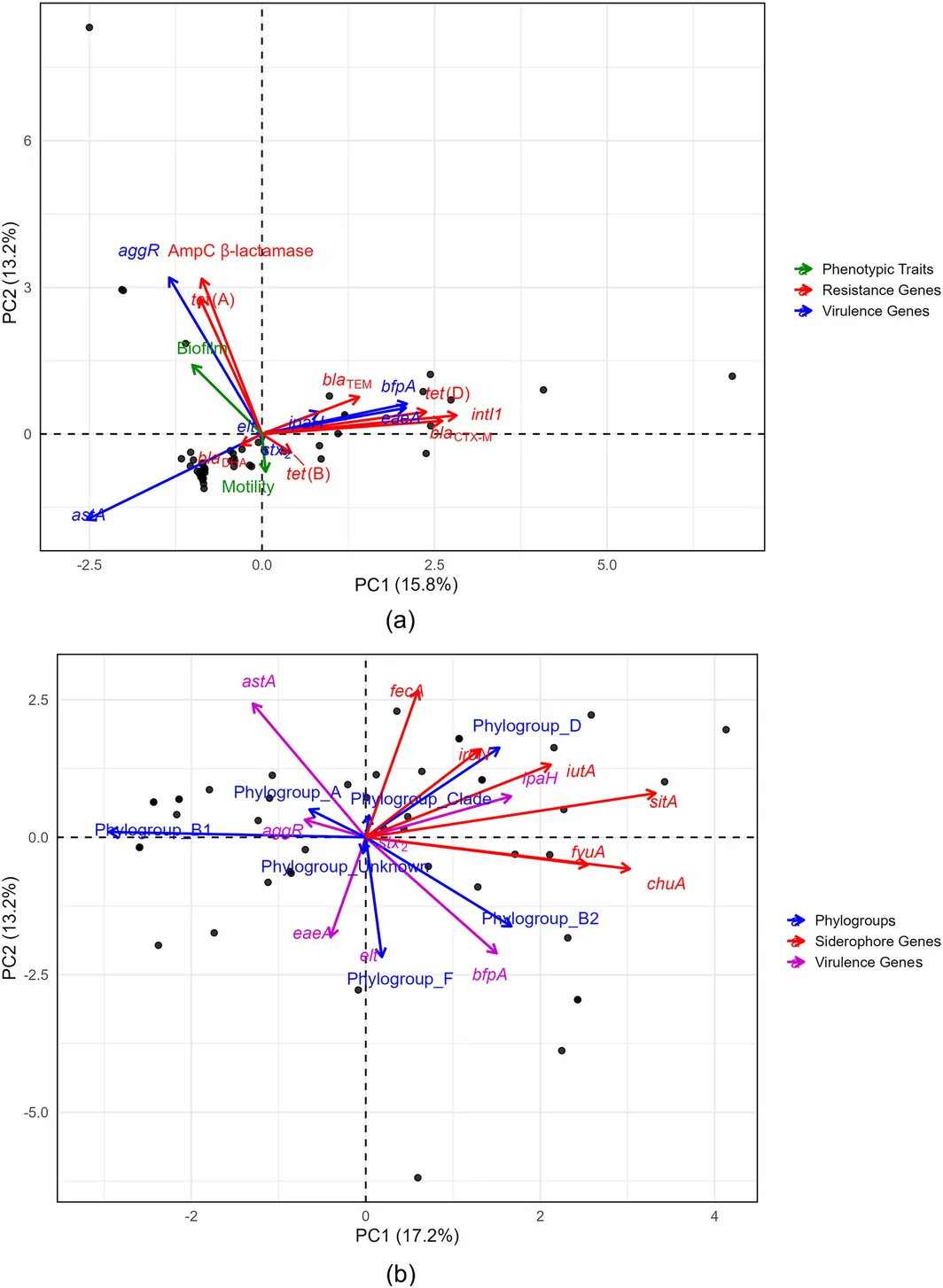
Principal component analysis (PCA) of genotypic and phenotypic characteristics of virulent *Escherichia coli* isolates. (a) PCA biplot showing associations among phenotypic traits (green), antimicrobial‐resistance determinants (red), and virulence genes (blue). PC1 and PC2 explained 15.8% and 13.2% of the total variance, respectively. (b) PCA biplot showing associations among phylogroup assignments (blue), siderophore genes (red), and virulence genes (magenta). PC1 and PC2 explained 17.2% and 13.2% of the total variance, respectively. Black points represent individual isolates. Vectors indicate variable loadings; their directions represent associations with the principal components, while their lengths indicate the relative contributions of the variables to the displayed components. Vectors pointing in similar directions indicate positive associations, whereas vectors pointing in opposite directions indicate negative associations. Dashed lines indicate the zero coordinates of the principal components.

Additional multivariate analyses of these relationships are presented in Figure 6. The gene–phenotype correlation network (Figure 6a) illustrated complex connectivity among traits, suggesting that several siderophore genes, particularly *chuA, fyuA*, and *sitA*, may play a central role in linking phylogroup, virulence, and resistance markers, while a small disconnected subnetwork indicated the presence of a distinct co‐occurrence module largely independent of the main network structure. In the PCA biplot (Figure 6b), a directional association was observed between biofilm formation and *aggR*, while motility exhibited an opposing pattern, consistent with the findings shown in Figure 5 and with CCA results. The CCA analysis (Figure 6c) indicated weak overall correlations between biofilm formation and most genetic traits, aligning with statistical tests showing no associations with siderophore genes, resistance genes, or phylogroups. Nevertheless, biofilm production differed significantly among some virulence‐gene groups. In contrast, motility showed a stronger canonical relationship with the examined genotypic traits than did biofilm formation, suggesting that motility may be more closely associated with genetic composition than biofilm formation, (Figure 6d).

**FIGURE 6:**
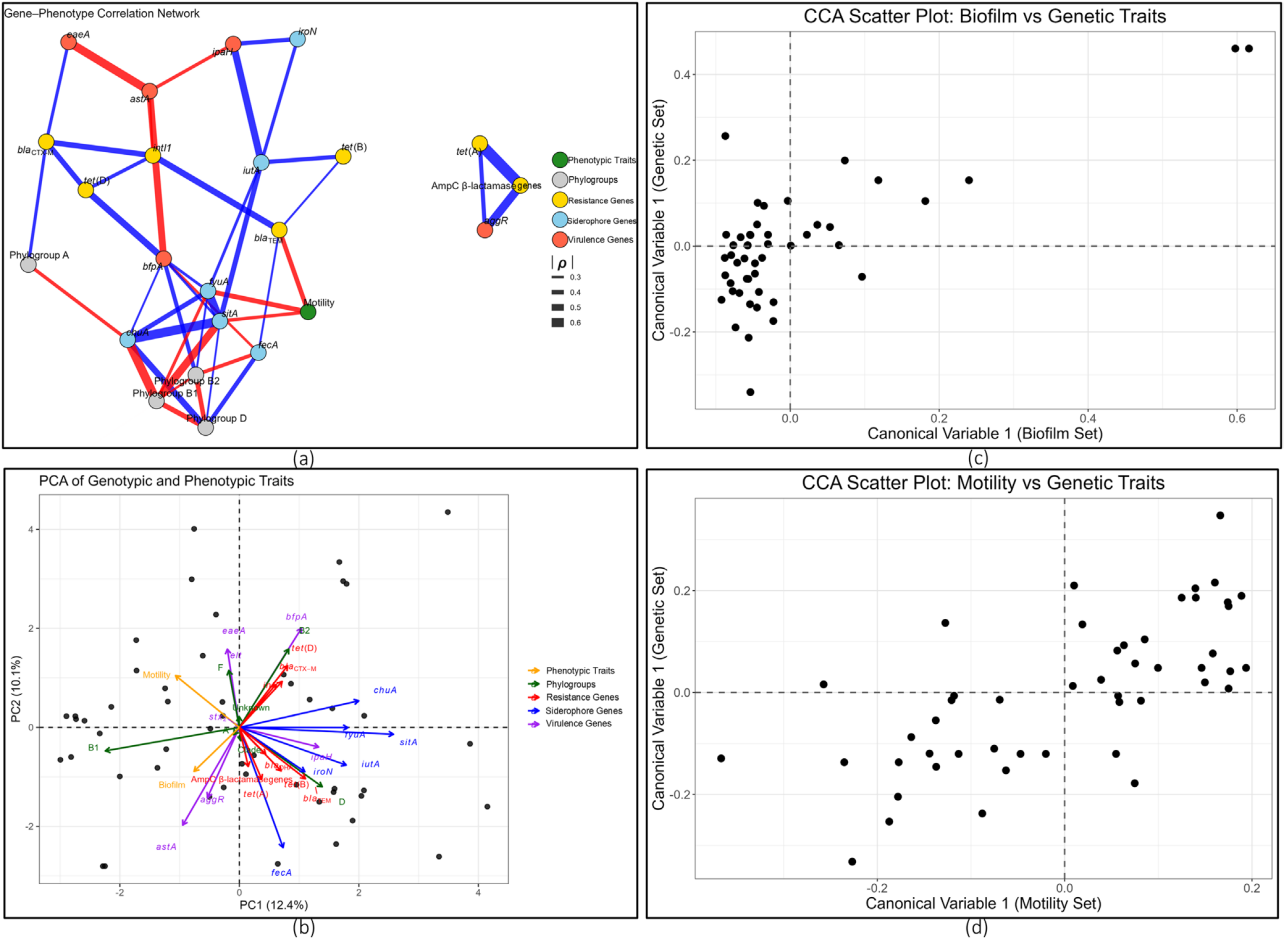
Integrated analysis of genotypic and phenotypic characteristics of virulent *Escherichia coli* isolates. (a) Spearman correlation network showing relationships among phenotypic traits, phylogroups, antimicrobial resistance determinants, siderophore genes, and virulence genes. Node colours indicate variable categories, as defined in the legend. Edge thickness represents the absolute Spearman correlation coefficient (|ρ|), while blue and red edges indicate positive and negative correlations, respectively. (b) Principal component analysis (PCA) biplot integrating phenotypic traits, phylogroups, antimicrobial resistance determinants, siderophore genes, and virulence genes. PC1 and PC2 explain 12.4% and 10.1% of the total variance, respectively. Black points represent individual isolates, and vectors indicate variable loadings. (c) Canonical correlation analysis (CCA) scores showing the relationship between biofilm formation and genetic characteristics. (d) CCA scores showing the relationship between motility and genetic characteristics. In panels (c) and (d), each point represents an individual isolate according to the first canonical variates of the phenotypic and genetic datasets. Dashed lines indicate zero on each axis.

## 4 Discussion

This study provides new insight into the integrated genotypic and phenotypic determinants that support the environmental persistence of *Escherichia coli* recovered from treated sewage in Japan. To our knowledge, this is the first study in Japan to comprehensively assess virulence determinants, AMR genes, siderophore systems, and environmental fitness traits in *E*. *coli* from multiple sewage treatment systems, extending previous studies that focused primarily on clinical isolates (Ohmura‐Hoshino et al. 2022; Zangane Matin et al. 2021) or a limited range of phenotypic characteristics (Zhi et al. 2019; Savin et al. 2021).

Despite being isolated from treated effluents, 13.70% of the *E*. *coli* strains harboured one or more virulence genes. This finding is consistent with reports from surface waters, in which virulence genes were detected in 24.7% and 27.0% of *E*. *coli* isolates by LaMontagne et al. (2023) and Morales‐Mora et al. (2025), respectively. The detection of virulence‐associated *E*. *coli* in treated effluents indicates that wastewater treatment processes may not completely eliminate strains carrying virulence determinants and highlights treated effluents as a potential route for the dissemination of these bacteria into downstream aquatic environments. In the present study, *astA* was the most frequently detected virulence gene. Strains carrying *astA* are classified as ODEC strains according to Kashima et al. (2021), and their high prevalence may reflect their ability to adapt to environmental reservoirs. Previous studies have documented the presence of *astA*‐positive *E*. *coli* in various sources, including patients with diarrhoea (Modgil et al. 2020; Ohmura‐Hoshino et al. 2022), caged and non‐caged retail table eggs (Sodagari et al. 2021), chicken livers (Gharaibeh et al. 2024), and raw milk and dairy products (Elbastawesy et al. 2025). Furthermore, an outbreak in Japan in 2021 demonstrated that *astA*‐positive *E*. *coli* strains could persist in food products (Kashima et al. 2021), underscoring their environmental resilience. The detection of *astA*‐positive *E*. *coli* in treated sewage effluents highlights the persistence of this virulence gene following wastewater treatment and demonstrates its continued occurrence in treated effluents.

The identification of hetero‐pathogenic pathotypes, such as aEPEC/ETEC and aEAEC/tEPEC, is of particular concern because the coexistence of virulence determinants from multiple pathotypes may enhance pathogenic potential and complicate clinical detection. Similar hetero‐pathogenic strains have been reported among clinical *E*. *coli* isolates, and their presence has been associated with increased disease severity (Santos et al. 2020; Finton et al. 2025). Furthermore, the higher prevalence of virulent isolates in combined sewer systems suggests that these systems may facilitate the environmental dissemination of pathogenic *E*. *coli* . Supporting this possibility, Zan et al. (2023) demonstrated that combined sewer overflow events contribute substantially to riverine bacterial communities during storm events. Together, these findings highlight the potential role of combined sewer systems as reservoirs and dissemination routes for virulence‐associated *E*. *coli* . However, no significant associations were detected between virulence status and season, Tsuyu (the Japanese rainy season), rainfall conditions, rainfall category, temperature, or precipitation amount. This suggests that the presence of virulent *E*. *coli* in treated effluent may be influenced more strongly by sewerage infrastructure and wastewater inputs than by short‐term weather‐related factors.

Resistance genes were identified in half of the virulence gene‐positive isolates, with *bla*_TEM_ representing the predominant determinant. Nevertheless, 50% of the isolates carrying virulence genes lacked detectable resistance genes, indicating that virulence and AMR do not necessarily coexist. This observation is consistent with previous studies reporting that not all virulent *E*. *coli* strains acquire AMR (Yazdanpour et al. 2020; Saini et al. 2024). Furthermore, no significant associations were observed between virulence genes and resistance genes, either globally or at the level of individual gene pairs. A similar pattern was reported by James et al. (2025), who found no significant association between virulence and AMR genes among multidrug‐resistant *E*. *coli* isolates from humans, animals, and environmental sources. Likewise, the prevalence of AMR genes did not differ significantly between isolates recovered from combined and separate sewer systems. These results indicate that AMR gene carriage was broadly distributed among virulence gene‐positive isolates and was not strongly associated with either virulence gene profiles or sewerage system type. In contrast, temporal analyses revealed significant variation in the prevalence of both virulence‐positive and AMR‐positive isolates, with 2023 showing the highest prevalence of both traits. This increase may reflect changes in wastewater inputs, microbial population dynamics, or environmental selection pressures, highlighting the importance of continued monitoring to better understand temporal variation in wastewater‐associated *E*. *coli* populations.

Phylogenetic analysis revealed that most virulence gene‐positive isolates belonged to phylogroup D, followed by B1. Although phylogroup B1 is commonly dominant in environmental *E*. *coli* populations and is considered well adapted to extra‐host environments (Moinet et al. 2021; Lagerstrom and Hadly 2023), phylogroup D predominated in the present study. Phylogroup D is frequently associated with ExPEC and virulence‐associated traits (Denamur et al. 2021). Similar observations have been reported among wastewater‐derived *E*. *coli* populations (Rodríguez Guevara et al. 2025). Together, these findings suggest that treated wastewater may harbour *E*. *coli* lineages possessing both environmental persistence and pathogenic potential. However, our study revealed no significant association between virulence genes and phylogenetic grouping. To date, few studies have investigated this relationship. These studies have been conducted primarily in clinical settings and have reported no significant differences in virulence factors across phylogenetic groups (Yazdanpour et al. 2020; Mahshouri et al. 2025; Van Bohemen et al. 2025). These findings underscore the dynamic nature of horizontal gene transfer and reinforce the need for expanded surveillance strategies that extend beyond traditional clinical lineages.

Siderophore systems play an important role in iron acquisition and bacterial fitness by enabling *E*. *coli* to thrive under iron‐limited conditions. However, in the present study, no significant differences were observed in the prevalence of siderophore genes between virulent and non‐virulent isolates, or between sewer system types. These findings suggest that iron acquisition systems are widely distributed among wastewater‐derived *E*. *coli* , regardless of virulence status. Among the accessory siderophore genes, *fecA* and *chuA* were the most prevalent. Both genes encode iron acquisition systems associated with bacterial fitness under iron‐limited conditions and have been reported in pathogenic and environmental *E*. *coli* populations (Frick‐Cheng et al. 2022; Krawczyk et al. 2025). Their high prevalence in the present study suggests that iron acquisition may contribute to the persistence of *E*. *coli* in treated wastewater environments.

In this study, no significant associations were observed between biofilm formation and either phylogenetic grouping or AMR profiles. Previous studies have reported inconsistent relationships among these traits, with some finding no significant associations (García et al. 2023; Mahshouri et al. 2025), whereas others have reported links between biofilm formation and specific phylogenetic groups or resistance determinants under particular conditions (Gunathilaka et al. 2024; Moniruzzaman et al. 2023). These discrepancies likely reflect differences in isolate origin, geographic region, sample size, and study design. Therefore, the biofilm‐forming capacity of wastewater‐derived *E*. *coli* may be influenced more by strain‐specific factors and environmental conditions than by phylogenetic background or AMR status alone.

In contrast, biofilm formation differed significantly among virulence gene groups, with *aggR*‐positive isolates exhibiting significantly greater biofilm production than the other virulence‐gene groups examined. The moderate effect size observed in the statistical analysis further supports the biological relevance of this association. The enhanced biofilm‐forming ability of *aggR*‐positive isolates is consistent with the established role of *aggR* as a central regulator of adherence and biofilm formation in EAEC (Benameur et al. 2021; Mickey and Nataro 2020). Enhanced surface colonization may contribute to the persistence of these strains in wastewater environments.

Motility assays revealed that all virulence gene‐positive *E*. *coli* isolates were motile, and that virulent isolates exhibited significantly greater motility than non‐virulent isolates. Although motility differed significantly among virulence‐gene groups, the effect size was small. Pairwise comparisons showed that *astA*‐, *bfpA*‐, and *eaeA*‐positive isolates exhibited greater motility than *ipaH*‐positive isolates. The association between *eaeA* and enhanced motility is supported by studies demonstrating that disruption of *eaeA* impairs Type III secretion system function, flagellar production, and motility in EPEC (Soria‐Bustos et al. 2022). The mechanisms underlying the associations of *astA* and *bfpA* with motility remain unclear and warrant further investigation.

Notably, no significant association was observed between motility and biofilm formation, suggesting that these phenotypic traits may be regulated independently. Nevertheless, *eaeA*‐ and *aggR*‐positive isolates exhibited a tendency toward an inverse biofilm–motility relationship. Although this trend did not reach statistical significance, Benyoussef et al. (2022) reported that highly motile *E*. *coli* cells exhibited reduced competitiveness during biofilm establishment, indicating that interactions between motility and biofilm formation may occur under certain conditions. Furthermore, Alshaikh et al. (2024) demonstrated that motility and biofilm production are governed by distinct genetic and regulatory pathways, supporting the notion that these traits can vary independently in response to environmental cues. Consistent with this interpretation, motility was not associated with phylogenetic groups, AMR genes, or siderophore‐related genes in the present study, a pattern also reported in previous investigations of environmental *E*. *coli* populations (Guerra et al. 2020; Wang et al. 2023). Collectively, these findings suggest that motility may represent an independent trait contributing to the ecological success and dissemination of wastewater‐derived *E*. *coli* .

The integration of genotypic and phenotypic data highlighted the multifactorial nature of adaptation in wastewater‐derived *E*. *coli* . The differentiation between motility—and biofilm‐associated traits suggests distinct ecological strategies, whereas the clustering of siderophore‐associated genes with specific phylogenetic backgrounds supports their role in lineage‐specific adaptation. Together, these findings indicate that environmental persistence is shaped by interactions among virulence‐associated traits, iron acquisition systems, and phenotypic fitness characteristics.

This study was limited to culturable isolates and may therefore underestimate the diversity of *E*. *coli* populations present in treated wastewater. Although temporal and seasonal analyses were performed, variation in sampling intensity among years may have influenced the observed temporal patterns. Future studies incorporating culture‐independent approaches and systematic longitudinal sampling are needed to provide a more comprehensive understanding of wastewater‐associated *E*. *coli* populations and their public health implications.

## 5 Conclusion

This study provides the first integrated genotypic and phenotypic assessment of *E*. *coli* isolates recovered from treated sewage in Japan. Virulence genes, particularly *astA*, together with the detection of hetero‐pathogenic pathotypes, the dominance of phylogroups D and B1, the widespread occurrence of *fecA* and *chuA*, and the frequent detection of *bla*_TEM_ highlight the adaptive potential of wastewater‐derived *E*. *coli* . The significant association between *aggR* and enhanced biofilm formation further suggests a role for virulence‐associated traits in environmental persistence. Collectively, these findings improve our understanding of the ecological significance of treated wastewater as a reservoir of potentially pathogenic *E*. *coli* and support the need for continued surveillance of wastewater effluents.

## Author Contributions

**Ida Munfarida:** investigation, formal analysis, data curation, visualization, writing – original draft. **Jant Cres Caigoy:** conceptualization, methodology, validation, writing – review and editing. **Christian Xedzro:** conceptualization, methodology, validation. **Toshi Shimamoto:** supervision, project administration, resources. **Tadashi Shimamoto:** conceptualization, supervision, writing – review and editing.

## Funding

This research was supported by Lembaga Pengelola Dana Pendidikan (LPDP; Indonesia Endowment Fund for Education), Ministry of Finance of the Republic of Indonesia, through the Beasiswa Indonesia Bangkit (BIB) scholarship program in collaboration with the Ministry of Religious Affairs (MoRA) of the Republic of Indonesia (Fellowship No. B‐446/Dt.I.III/PP.04/02/2024).

## Conflicts of Interest

The authors declare no conflicts of interest.

## Supporting information


**Table S1:** Primer sequences used for the detection of diarrheagenic *Escherichia coli* (DEC) pathotypes. The table lists forward and reverse primer sequences, along with their target genes and corresponding amplicon sizes, used for multiplex PCR assays to identify specific DEC virulence markers.
**Table S2:** (a) Group‐specific oligonucleotides used in this study for eicosaplex PCR. (b) Group‐specific oligonucleotides used in this study for octaplex PCR. (c) Primers used for the detection of *mcr‐1*, *mcr‐2*, *mcr‐3*, *mcr‐4* and *mcr‐5* genes by multiplex PCR. (d) Primer and PCR products for the *mcr‐6* to *mcr‐9* multiplex PCR assay. (e) Primer sequences used for the amplification of *mcr‐10*. (f) Primer sequences used for the amplification of tetracyline‐resistant gene.
**Table S3:** (a) Primer sequences, target genes, and expected amplicon sizes used in the extended quadruplex PCR method for *Escherichia coli* phylo‐group. (b) Quadruplex PCR genotypes and corresponding steps for phylo‐group assignment of *Escherichia coli* isolates using the extended Clermont method determination.
**Table S4:** Primer Sequences and PCR Conditions for Detection of Siderophore‐Associated Genes in *Escherichia coli* .
**Table S5:** (a) Sampling, geographic, and meteorological data for *Escherichia coli* isolates. (b) Seasonal distribution of recovered *Escherichia coli* isolates. (c) Distribution of *Escherichia coli* isolates by Tsuyu status. (d) Distribution of *Escherichia coli* isolates by rainfall category.
**Table S6:** Summary of *E*. *coli* isolate counts by sampling date, location, and sewage treatment system. A total of 365 isolates were recovered. Kruskal–Wallis tests indicated significant variation in isolate recovery among sampling locations (*p* = 0.0026), with Asahimachi and Eba showing higher isolate counts than Higashihiroshima and Tobu. Isolate recovery also differed significantly between sewage treatment systems (*p* = 0.0013).
**Table S7:** (a) Sampling details and detected virulence genes of *Escherichia coli* isolates. (b) Distribution of virulent and non‐virulent *Escherichia coli* isolates across seasons, rainfall conditions, and meteorological variables. (c) Distribution of virulence gene‐positive *Escherichia coli* isolates by sampling date, location, and sewage treatment system. Significant differences were observed between sewage treatment system types (χ^2^ = 23.12, *p* < 0.001) and among sampling locations (*χ* ^2^ = 22.96, *p* < 0.001). (d) Year‐versus‐all‐other‐years comparisons of virulence‐positive *Escherichia coli* isolates recovered from treated wastewater. Temporal differences were evaluated using Fisher's exact tests, followed by Benjamini–Hochberg (BH) correction for multiple comparisons. Significant adjusted *p* values (< 0.05) indicate years in which the prevalence of virulence‐positive isolates differed significantly from that observed across all other sampling years combined.
**Table S8:** (a) Comparison of resistance gene carriage between virulence gene‐positive *Escherichia coli* isolates recovered from combined and separate sewer systems. No significant association was observed between resistance gene carriage and sewer system type (Fisher's exact test, *p* = 0.702). (b) Year‐versus‐all‐other‐years comparisons of antimicrobial resistance (AMR)‐positive *Escherichia coli* isolates recovered from treated wastewater. Temporal differences were evaluated using Fisher's exact tests, followed by Benjamini–Hochberg (BH) correction for multiple comparisons. Significant adjusted *p* values (< 0.05) indicate years in which the prevalence of AMR‐positive isolates differed significantly from that observed across all other sampling years combined. (c) Comparison of siderophore gene carriage between virulence gene‐positive *Escherichia coli* isolates recovered from combined and separate sewer systems. No significant association was observed between siderophore gene carriage and sewer system type (Fisher's exact test, *p* = 1).
**Table S9:** (a) Composition of *E*. *coli* Phylogroups Recovered from Sewage Treatment Water. (b) Distribution of Virulence Genes among *E*. *coli* Phylogroups Isolated from Treated Sewage Water. (c) Distribution of *Escherichia coli* isolates by virulence gene and phylogroup. Fisher's exact test with Monte Carlo simulation assessing the association between virulence gene profiles and phylogenetic groups of virulent *Escherichia coli* isolates. No significant association was detected (*p* = 0.6014).
**Table S10:** (a) In silico detection of virulence and iron acquisition genes in 50 *E*. *coli* isolates retrieved from the EnteroBase. (b) Prevalence of virulence genes in *Escherichia coli* isolates by source niche, with associated *p* values, Cramér's V effect sizes, and significance labels based on Chi‐squared or Fisher's exact tests. (c) Prevalence of siderophore genes in *Escherichia coli* isolates by source niche, with associated *p* values, Cramér's V effect sizes, and significance labels based on Chi‐squared or Fisher's exact tests.
**Table S11:** Biofilm formation ability of 50 *Escherichia coli* isolates based on average OD₅₇₀ values measured in a microtiter plate assay.
**Table S12:** Univariate analysis of the association between biofilm formation and the presence of antibiotic resistance genes, siderophore genes, and phylogenetic groups in *Escherichia coli* isolates. *p* values were calculated using the Wilcoxon Rank‐Sum Test. Adjusted *p* values were corrected for multiple comparisons using the False Discovery Rate (FDR) method. No associations remained statistically significant after adjustment (adjusted *p* > 0.05).
**Table S13:** Motility assessment of *Escherichia coli* isolates based on average swimming diameter in soft agar.
**Table S14:** (a) Univariate analysis of the association between motility and the presence of antibiotic resistance genes, siderophore genes, and phylogenetic groups in *Escherichia coli* isolates. *p* values were calculated using the Wilcoxon Rank‐Sum Test. Adjusted *p* values were obtained using the False Discovery Rate (FDR) method. (b) Spearman correlation between biofilm formation and motility in *E*. *coli* isolates, showing no statistically significant association (*p* > 0.05). Although eaeA—and aggR‐positive isolates showed a tendency toward an inverse biofilm–motility relationship.

**Figure S1:** PCR confirmation of *Escherichia coli* isolates using the Ecuid primer set targeting the *uidA* gene (expected amplicon size: 171 bp). Lane L, 100‐bp DNA ladder; lane P, positive control ( *E*. *coli* ETEC strain 0509271); lane N, negative control ( *Salmonella enterica* serovar Typhimurium strain LT2). Lanes 1–20 correspond to isolates A‐2‐5b, A‐2‐5c, A‐2‐8a, A‐2‐8b, A‐2‐8c, A‐2‐17, A‐2‐19a, A‐2‐19b, A‐2‐19c, E‐2‐1a, E‐2‐1b, E‐2‐2, E‐2‐5, E‐2‐9a, E‐2‐9b, E‐2‐17a, E‐2‐17b, E‐2‐19a, E‐2‐19b, and E‐2‐19c, respectively.
**Figure S2:** (a) Detection of 10 virulence genes in reference *E*. *coli* strains using multiplex PCR to establish a comprehensive panel of positive controls. Lane L, 100‐bp DNA ladder; lanes 1–14 correspond to *E*. *coli* strains: ETEC 0509266, ETEC 0509267, ETEC 0509270, ETEC 0509271, ETEC 0509274, ETEC 0509276, EHEC RIMD 0509891, EHEC RIMD 0509894, EHEC RIMD 0509929, EHEC RIMD 0509825, EHEC RIMD 05091053, EPEC 0509829, EAEC 0591633, and EIEC 0591045, respectively. The gel shows amplification of *astA, aggR, bfpA, eaeA, elt, invE, ipaH, stx*_*1*_, *stx*_*2*_, and *sth* genes. Based on these results, a mixture of genomic DNA from selected positive strains was used as a multiplex positive control in subsequent assays: strain ETEC 0509271 (*astA, sth, elt*), EHEC RIMD 0509891 (*astA, eaeA, stx*_*1*_, *stx*_*2*_), EPEC 0509829 (*bfpA, eaeA*), EAEC 0591633 (*astA, aggR*), and EIEC 0591045 (*invE, ipaH*). (b) Confirmation of virulence gene presence in *E*. *coli* positive control isolates using single PCR. Lanes 1–14 correspond to ETEC 0509266, ETEC 0509267, ETEC 0509270, ETEC 0509271, ETEC 0509274, ETEC 0509276, EHEC RIMD 0509891, EHEC RIMD 0509894, EHEC RIMD 0509929, EHEC RIMD 0509825, EHEC RIMD 05091053, EPEC 0509829, EAEC 0591633, and EIEC 0591045, respectively. PCR results validated the findings from multiplex assays. Specific bands indicate gene presence: *sth* (Lane 1), *bfpA* (Lane 4), *aggR* (Lane 5), *elt* (Lanes 6–7), *ipaH* (Lane 8) and *eaeA* (Lanes 9–14). (c) Confirmation of virulence gene presence in *E*. *coli* positive control isolates using single PCR. Lanes 1–14 correspond to ETEC 0509266, ETEC 0509267, ETEC 0509270, ETEC 0509271, ETEC 0509274, ETEC 0509276, EHEC RIMD 0509891, EHEC RIMD 0509894, EHEC RIMD 0509929, EHEC RIMD 0509825, EHEC RIMD 05091053, EPEC 0509829, EAEC 0591633, and EIEC 0591045, respectively. PCR results validated the findings from multiplex assays. Specific bands indicate gene presence: *invE* (Lane 7), and *astA* (Lanes 8–14). (d) Confirmation of virulence gene presence in *E*. *coli* positive control isolates using single PCR. Lanes 1–5 correspond to strains EHEC RIMD 0509891, EHEC RIMD 0509894, EHEC RIMD 0509929, EHEC RIMD 0509825, EHEC RIMD 05091053 respectively. PCR results validated the findings from multiplex assays. Specific bands indicate gene presence: *stx*_*2*_ and *stx*_*1*_ in all tested isolates. (e) Multiplex PCR results for the detection of virulence genes in *E*. *coli* isolates. Lane P, positive control; lanes 1–20, tested isolates (in order: ⑩‐N‐1C‐B, ⑩‐N‐2C‐C, ⑩‐N‐4C‐B, ⑩‐N‐4C‐C, ⑩‐N‐4C‐D, ⑩‐N‐5C‐A, ⑩‐N‐2CA‐A, ⑩‐N‐3CA‐A, ⑩‐T‐1CA‐B, E‐11, E‐13, E‐17, E‐21, E‐2‐Aa, E‐3‐A, E‐5, E‐9, H‐10, S‐14, S‐16); lane N, negative control. Distinct bands indicate the presence of specific virulence genes. Notably, bands of the expected sizes for *aggR, astA*, and *ipaH* were observed in lanes 9; 6, 9, and 14; and 6, 10, 14, 15, and 20, respectively. These results were subsequently verified by single PCR. (f) Single PCR confirmation of *astA* gene following multiplex PCR screening. Lane L,100‐bp DNA ladder; lane P, positive control for *astA*; lanes 1–11, *E*. *coli* isolates E‐1, ②‐E‐3CA‐A, ②‐E‐3CA‐B, ④‐S‐1CA‐B, ④‐S‐2CA‐A, ⑦‐E‐1C‐C, ⑨‐E‐2CA‐C, ⑩‐T‐1CA‐A, S‐2‐21, E‐2‐B‐c, and E‐3‐A. The gel shows that all isolates were positive for the *astA* gene. (g) Single PCR confirmation of *ipaH* gene following multiplex PCR screening. Lane L, 100‐bp DNA ladder; lane P, positive control for *ipaH*; lanes 1–5, *E*. *coli* isolates ➂‐A‐4CA, ➂‐N‐2CA, ➂‐N‐MP, ➅‐N‐1‐CAa, and ➅‐N‐1‐CAb. Lane 3 showed a positive band corresponding to the *ipaH* gene.
**Figure S3:** Eicosa‐Plex PCR detection of antimicrobial resistance‐related markers in *Escherichia coli* isolates. Lane L, 100‐bp DNA ladder; lane P, positive control for the Eicosa‐Plex assay; lanes 1–20, isolates ②‐E‐4CA‐Ad, ➂‐N‐MP, ④‐S‐1CA‐Bb, ④‐S‐1CA‐Bc, ➃‐S‐4CA‐Bb, ➃‐S‐4CA‐Bc, ⑥‐A‐1CA‐Da, ⑥‐A‐1CA‐Db, ⑥‐A‐3CA‐Aa, ⑥‐N‐2CA, ⑦‐S‐4C‐B, ⑦‐S‐5C‐Aa, ⑦‐S‐5C‐Ab, ⑦‐S‐5C‐B, ➆‐E‐2C‐Cb, ➆‐E‐2C‐Cc, ⑦‐E‐4C‐Bc, ⑦‐E‐4C‐Ca, ⑧‐N‐4C‐A, and E‐2‐20b, respectively; lane N, negative control. Amplicons corresponding to *bla*_TEM_ were detected in lanes 1–9; *bla*_CTX‐M_ in lanes 5, 6, and 10; AmpC β‐lactamase genes in lanes 7 and 8; and *intI1* in lanes 1 and 4–6.
**Figure S4:** Phylogroup determination of *Escherichia coli* isolates using quadruplex PCR. Lane L, 100‐bp DNA ladder; lane P1, positive control for phylogroup B2 ( *E*. *coli* ATCC 25922); lane P2, positive control for phylogroup B1 ( *E*. *coli* strain 198); lane N, negative control. Lanes 1–18 correspond to isolates ②‐E‐4CA‐Ad, ➂‐N‐MP, ④‐S‐1CA‐Bb, ④‐S‐1CA‐Bc, ➃‐S‐4CA‐Bb, ➃‐S‐4CA‐Bc, ⑥‐A‐1CA‐Da, ⑥‐A‐1CA‐Db, ⑥‐A‐3CA‐Aa, ⑥‐N‐2CA, ⑦‐S‐4C‐B, ⑦‐S‐5C‐Aa, ⑦‐S‐5C‐Ab, ⑦‐S‐5C‐B, ➆‐E‐2C‐Cb, ➆‐E‐2C‐Cc, ⑦‐E‐4C‐Bc, and ⑦‐E‐4C‐Ca, respectively. Based on the quadruplex PCR banding patterns, lanes 1, 3, and 7–9 showed the D/E profile; lanes 2 and 10–14 showed the B2 profile; lanes 4 and 15–18 showed the B1 profile; and lanes 5 and 6 showed the A/C profile.
**Figure S5:** (a) Multiplex PCR detection of the siderophore‐associated genes *iroN*, *iutA*, and *fecA* in *Escherichia coli* isolates. Lane L, 100‐bp DNA ladder; lane P, positive control ( *E*. *coli* strain ④‐S‐1CA‐Ba, previously confirmed to carry all three target genes); lane N, negative control ( *E*. *coli* strain MA‐1‐1‐b, previously confirmed to be negative for all three target genes). Lanes 1–20 correspond to isolates ②‐E‐4CA‐Ad, ➂‐N‐MP, ④‐S‐1CA‐Bb, ④‐S‐1CA‐Bc, ➃‐S‐4CA‐Bb, ➃‐S‐4CA‐Bc, ⑥‐A‐1CA‐Da, ⑥‐A‐1CA‐Db, ⑥‐A‐3CA‐Aa, ⑥‐N‐2CA, ⑦‐S‐4C‐B, ⑦‐S‐5C‐Aa, ⑦‐S‐5C‐Ab, ⑦‐S‐5C‐B, ➆‐E‐2C‐Cb, ➆‐E‐2C‐Cc, ⑦‐E‐4C‐Bc, ⑦‐E‐4C‐Ca, ⑧‐N‐4C‐A, and ⑧‐N‐4C‐Ab, respectively. Bands at the expected amplicon sizes indicate the presence of the corresponding siderophore‐associated genes. (b) Single PCR confirmation of *fecA* following multiplex PCR screening. Lane L, 100‐bp DNA ladder; lane P, positive control ( *E*. *coli* strain ④‐S‐1CA‐Ba); lane N, negative control ( *E*. *coli* strain MA‐1‐1‐b). Lanes 1–20 correspond to isolates ②‐E‐3CA‐A, ②‐E‐3CA‐B, ②‐E‐4CA‐Aa, ②‐E‐4CA‐Ab, ②‐E‐4CA‐Ac, ④‐S‐1CA‐Bb, ④‐S‐2CA‐A, ⑧‐A‐1CA‐C, ⑧‐A‐5CA‐C, ⑨‐E‐2CA‐C, ⑩‐T‐1CA‐A, E‐1, E‐3‐A, S‐2‐21, E‐2‐24a, E‐2‐24b, E‐2‐24c, A‐2‐17, A‐2‐19a, and A‐2‐19b, respectively. All tested isolates showed amplification of *fecA* at the expected amplicon size.

## Data Availability

The data that support the findings of this study are available in the Supporting Information of this article.
