# Supplementary material for: Integrated Genotypic and Phenotypic Determinants Underpin the Environmental Persistence of Escherichia coli in Treated Sewage in Japan
Source: Environ Microbiol Rep. 2026 Oct 30;18(6):e70452. doi: 10.1111/1758-2229.70452 (PMC13722885; doi:10.1111/1758-2229.70452)
Supplement: Supplementary file 1 [file EMI4-18-e70452-s002.docx]

**Supplementary information for “Integrated genotypic and phenotypic determinants underpin the environmental persistence of *Escherichia coli* in treated sewage in Japan”**

Ida Munfarida^a,b^, Jant Cres Caigoy^a^, Christian Xedzro^a#^, Toshi Shimamoto^a^, Tadashi Shimamoto^a^*

^a^Graduate School of Integrated Sciences for Life, Hiroshima University, 1-4-4 Kagamiyama, Higashi-Hiroshima, Hiroshima 739-8528, Japan

^b^Faculty of Science and Technology, Universitas Islam Negeri Sunan Ampel Surabaya, Jl. Dr. Ir. Soekarno No. 682, Gunung Anyar, Surabaya 60294, Indonesia

^#^Present affiliation: Division of Anti-Infective Drug Discovery, Center for Infectious Disease Research, Fujita Health University, 1-98 Dengakugakubo, Kutsukake-cho, Toyoake, 470-1192, Japan

Email: [d246872@hiroshima-u.ac.jp](mailto:d246872@hiroshima-u.ac.jp), [jccaigoy@hiroshima-u.ac.jp](mailto:jccaigoy@hiroshima-u.ac.jp), christian.xedzro@fujita-hu.ac.jp, [tsima@hiroshima-u.ac.jp](mailto:tsima@hiroshima-u.ac.jp), [tadashis@hiroshima-u.ac.jp](mailto:tadashis@hiroshima-u.ac.jp)

*Correspondence: Tadashi Shimamoto

Graduate School of Integrated Sciences for Life, Hiroshima University, 1-4-4 Kagamiyama, Higashi-Hiroshima, Hiroshima 739-8528, Japan

Email: [tadashis@hiroshima-u.ac.jp](mailto:tadashis@hiroshima-u.ac.jp)

**Figure S1.** PCR confirmation of Escherichia coli isolates using the Ecuid primer set targeting the *uidA* gene (expected amplicon size: 171 bp). Lane L, 100-bp DNA ladder; lane P, positive control (*E. coli* ETEC strain 0509271); lane N, negative control (*Salmonella enterica* serovar Typhimurium strain LT2). Lanes 1–20 correspond to isolates A-2-5b, A-2-5c, A-2-8a, A-2-8b, A-2-8c, A-2-17, A-2-19a, A-2-19b, A-2-19c, E-2-1a, E-2-1b, E-2-2, E-2-5, E-2-9a, E-2-9b, E-2-17a, E-2-17b, E-2-19a, E-2-19b, and E-2-19c, respectively.

**(a)**

**(b)**

**(c)**

**(d)**

**(e)**

**(f)	(g)**

**Figure S2.** (a) Detection of 10 virulence genes in reference *E. coli* strains using multiplex PCR to establish a comprehensive panel of positive controls. Lane L, 100-bp DNA ladder; lanes 1–14 correspond to *E. coli* strains*:* ETEC 0509266, ETEC 0509267, ETEC 0509270, ETEC 0509271, ETEC 0509274, ETEC 0509276, EHEC RIMD 0509891, EHEC RIMD 0509894, EHEC RIMD 0509929, EHEC RIMD 0509825, EHEC RIMD 05091053, EPEC 0509829, EAEC 0591633, and EIEC 0591045, respectively. The gel shows amplification of *astA, aggR, bfpA, eaeA, elt, invE, ipaH, stx_1_, stx_2_,* and *sth* genes. Based on these results, a mixture of genomic DNA from selected positive strains was used as a multiplex positive control in subsequent assays: strain ETEC 0509271 (*astA, sth, elt*), EHEC RIMD 0509891 (*astA, eaeA, stx_1_, stx_2_*), EPEC 0509829 (*bfpA, eaeA*), EAEC 0591633 (*astA, aggR*), and EIEC 0591045 (*invE, ipaH*). (b) Confirmation of virulence gene presence in *E. coli* positive control isolates using single PCR. Lanes 1–14 correspond to ETEC 0509266, ETEC 0509267, ETEC 0509270, ETEC 0509271, ETEC 0509274, ETEC 0509276, EHEC RIMD 0509891, EHEC RIMD 0509894, EHEC RIMD 0509929, EHEC RIMD 0509825, EHEC RIMD 05091053, EPEC 0509829, EAEC 0591633, and EIEC 0591045, respectively. PCR results validated the findings from multiplex assays. Specific bands indicate gene presence: *sth* (Lane 1), *bfpA* (Lane 4), *aggR* (Lane 5), *elt* (Lanes 6–7), *ipaH* (Lane 8) and *eaeA* (Lanes 9–14). (c) Confirmation of virulence gene presence in *E. coli* positive control isolates using single PCR. Lanes 1–14 correspond to ETEC 0509266, ETEC 0509267, ETEC 0509270, ETEC 0509271, ETEC 0509274, ETEC 0509276, EHEC RIMD 0509891, EHEC RIMD 0509894, EHEC RIMD 0509929, EHEC RIMD 0509825, EHEC RIMD 05091053, EPEC 0509829, EAEC 0591633, and EIEC 0591045, respectively. PCR results validated the findings from multiplex assays. Specific bands indicate gene presence: *invE* (Lane 7), and *astA* (Lanes 8-14). (d) Confirmation of virulence gene presence in *E. coli* positive control isolates using single PCR. Lanes 1–5 correspond to strains EHEC RIMD 0509891, EHEC RIMD 0509894, EHEC RIMD 0509929, EHEC RIMD 0509825, EHEC RIMD 05091053 respectively. PCR results validated the findings from multiplex assays. Specific bands indicate gene presence: *stx_2_* and *stx_1_* in all tested isolates. (e) Multiplex PCR results for the detection of virulence genes in *E. coli* isolates. Lane P, positive control; lanes 1–20, tested isolates (in order: ⑩-N-1C-B, ⑩-N-2C-C, ⑩-N-4C-B, ⑩-N-4C-C, ⑩-N-4C-D, ⑩-N-5C-A, ⑩-N-2CA-A, ⑩-N-3CA-A, ⑩-T-1CA-B, E-11, E-13, E-17, E-21, E-2-Aa, E-3-A, E-5, E-9, H-10, S-14, S-16); lane N, negative control. Distinct bands indicate the presence of specific virulence genes. Notably, bands of the expected sizes for *aggR, astA,* and *ipaH* were observed in lanes 9; 6, 9, and 14; and 6, 10, 14, 15, and 20, respectively. These results were subsequently verified by single PCR. (f) Single PCR confirmation of *astA* gene following multiplex PCR screening. Lane L,100-bp DNA ladder; lane P, positive control for *astA*; lanes 1–11, *E. coli* isolates E-1, ②-E-3CA-A, ②-E-3CA-B, ④-S-1CA-B, ④-S-2CA-A, ⑦-E-1C-C, ⑨-E-2CA-C, ⑩-T-1CA-A, S-2-21, E-2-B-c, and E-3-A. The gel shows that all isolates were positive for the *astA* gene. (g) Single PCR confirmation of *ipaH* gene following multiplex PCR screening. Lane L, 100-bp DNA ladder; lane P, positive control for *ipaH*; lanes 1–5, *E. coli* isolates ➂-A-4CA, ➂-N-2CA, ➂-N-MP, ➅-N-1-CAa, and ➅-N-1-CAb. Lane 3 showed a positive band corresponding to the *ipaH* gene.

**Figure S3.** Eicosa-Plex PCR detection of antimicrobial resistance-related markers in *Escherichia coli* isolates. Lane L, 100-bp DNA ladder; lane P, positive control for the Eicosa-Plex assay; lanes 1–20, isolates ②-E-4CA-Ad, ➂-N-MP, ④-S-1CA-Bb, ④-S-1CA-Bc, ➃-S-4CA-Bb, ➃-S-4CA-Bc, ⑥-A-1CA-Da, ⑥-A-1CA-Db, ⑥-A-3CA-Aa, ⑥-N-2CA, ⑦-S-4C-B, ⑦-S-5C-Aa, ⑦-S-5C-Ab, ⑦-S-5C-B, ➆-E-2C-Cb, ➆-E-2C-Cc, ⑦-E-4C-Bc, ⑦-E-4C-Ca, ⑧-N-4C-A, and E-2-20b, respectively; lane N, negative control. Amplicons corresponding to *bla*_TEM_ were detected in lanes 1–9; *bla*_CTX-M_ in lanes 5, 6, and 10; AmpC β-lactamase genes in lanes 7 and 8; and *intI1* in lanes 1 and 4–6.

**Figure S4.** Phylogroup determination of *Escherichia coli* isolates using quadruplex PCR. Lane L, 100-bp DNA ladder; lane P1, positive control for phylogroup B2 (*E. coli* ATCC 25922); lane P2, positive control for phylogroup B1 (*E. coli* strain 198); lane N, negative control. Lanes 1–18 correspond to isolates ②-E-4CA-Ad, ➂-N-MP, ④-S-1CA-Bb, ④-S-1CA-Bc, ➃-S-4CA-Bb, ➃-S-4CA-Bc, ⑥-A-1CA-Da, ⑥-A-1CA-Db, ⑥-A-3CA-Aa, ⑥-N-2CA, ⑦-S-4C-B, ⑦-S-5C-Aa, ⑦-S-5C-Ab, ⑦-S-5C-B, ➆-E-2C-Cb, ➆-E-2C-Cc, ⑦-E-4C-Bc, and ⑦-E-4C-Ca, respectively. Based on the quadruplex PCR banding patterns, lanes 1, 3, and 7–9 showed the D/E profile; lanes 2 and 10–14 showed the B2 profile; lanes 4 and 15–18 showed the B1 profile; and lanes 5 and 6 showed the A/C profile.

**(a)**

**(b)**

**Figure S5.** (a) Multiplex PCR detection of the siderophore-associated genes *iroN*, *iutA*, and *fecA* in *Escherichia coli* isolates. Lane L, 100-bp DNA ladder; lane P, positive control (*E. coli* strain ④-S-1CA-Ba, previously confirmed to carry all three target genes); lane N, negative control (*E. coli* strain MA-1-1-b, previously confirmed to be negative for all three target genes). Lanes 1–20 correspond to isolates ②-E-4CA-Ad, ➂-N-MP, ④-S-1CA-Bb, ④-S-1CA-Bc, ➃-S-4CA-Bb, ➃-S-4CA-Bc, ⑥-A-1CA-Da, ⑥-A-1CA-Db, ⑥-A-3CA-Aa, ⑥-N-2CA, ⑦-S-4C-B, ⑦-S-5C-Aa, ⑦-S-5C-Ab, ⑦-S-5C-B, ➆-E-2C-Cb, ➆-E-2C-Cc, ⑦-E-4C-Bc, ⑦-E-4C-Ca, ⑧-N-4C-A, and ⑧-N-4C-Ab, respectively. Bands at the expected amplicon sizes indicate the presence of the corresponding siderophore-associated genes. (b) Single PCR confirmation of *fecA* following multiplex PCR screening. Lane L, 100-bp DNA ladder; lane P, positive control (*E. coli* strain ④-S-1CA-Ba); lane N, negative control (*E. coli* strain MA-1-1-b). Lanes 1–20 correspond to isolates ②-E-3CA-A, ②-E-3CA-B, ②-E-4CA-Aa, ②-E-4CA-Ab, ②-E-4CA-Ac, ④-S-1CA-Bb, ④-S-2CA-A, ⑧-A-1CA-C, ⑧-A-5CA-C, ⑨-E-2CA-C, ⑩-T-1CA-A, E-1, E-3-A, S-2-21, E-2-24a, E-2-24b, E-2-24c, A-2-17, A-2-19a, and A-2-19b, respectively. All tested isolates showed amplification of *fecA* at the expected amplicon size.
